# Supplementary material for: Consensus on the descriptors, definitions, and reporting methods for heading in football studies: A Delphi study
Source: PLoS One. 2025 Jul 17;20(7):e0327189. doi: 10.1371/journal.pone.0327189 (PMC12270182; doi:10.1371/journal.pone.0327189)
Supplement: S1 Appendix — (DOCX) [file pone.0327189.s001.docx]

# Consensus on the descriptors, definitions, and reporting methods for heading in football studies: A Delphi study

# Supplementary I Appendix

This appendix formed part of the original submission for peer review.

This appendix contains additional project material as well as all response data from the 5 questionnaires.

Contents

[**Consensus on the descriptors, definitions, and reporting methods for heading in football studies: A Delphi study** 1](#_Toc190357541)

[Supplementary Appendix I 1](#_Toc190357542)

[Heading In Football Project Steering Committee 3](#_Toc190357543)

[Authors and affiliations: 3](#_Toc190357544)

[Expertise: 4](#_Toc190357545)

[Table 1: Demographics of the Project Steering Committee 4](#_Toc190357546)

[Respondents who agreed to be named and acknowledged for their participation. 6](#_Toc190357547)

[List of studies used to inform heading descriptors: 9](#_Toc190357548)

[Invitees including responders and non-responders 14](#_Toc190357549)

[Table 2: Invitees including responders and non-responders 14](#_Toc190357550)

[Results 29](#_Toc190357553)

[Phase 1- First Round Questionnaire 29](#_Toc190357554)

[Table 3: Results for Phase 1- first-round questionnaire for all participants and per role (%) 30](#_Toc190357555)

[Phase 1- Second-Round Questionnaire 32](#_Toc190357556)

[Table 4: Agreement results for Phase 1 - first-round questionnaire for all participants and per role (%) 33](#_Toc190357557)

[Phase 2- First-Round Questionnaire 35](#_Toc190357558)

[Table 5: Agreement results Phase 2- first round questionnaire for all participants and per role (%). 36](#_Toc190357559)

[Phase 2- Phase 2- Second Round Questionnaire 41](#_Toc190357560)

[Table 6: Agreement results Phase 2 - second-round questionnaire for all participants and per role (%). 42](#_Toc190357561)

[Phase 3- First round questionnaire 45](#_Toc190357562)

[Table 7: Completion of Phase 3 questionnaire for all participants and per role (%) 45](#_Toc190357563)

# Heading In Football Project Steering Committee

## Authors and affiliations:

Kerry Peek,^1,2^ Andrew Ross,^1,3^ Paula R Williamson,^4^ Julia Georgieva,^1,5^ Thor Einar Andersen,^6,7^ Tim Meyer,^8^ Vincent Gouttebarge,^9,10,11^ Sara Dahlen,^6^ Mike Clarke,^12^ Andreas Serner^2^

^1^ Sydney School of Health Sciences, Faculty of Medicine and Health, The University of Sydney. Australia.

^2^ FIFA Medical, Fédération Internationale de Football Association, Zurich, Switzerland

^3^ Physiotherapy Department, College of Sport, Health and Engineering, Victoria University, Melbourne, Australia.

^4^ Department of Health Data Science, University of Liverpool, UK

**^5^** Curtin School of Allied Health, Curtin University, Perth, WA, 6102, Australia

^6^ Oslo Sports Trauma Research Center, the Norwegian School of Sport Sciences, Oslo, Norway

^7^ The Norwegian Football Association’s Sports Medical Centre, Oslo, Norway

^8^ Institute of Sports and Preventive Medicine, Saarland University, Germany

^9^ Amsterdam UMC location University of Amsterdam, Department of Orthopedic Surgery and Sports Medicine, Meibergdreef 9, Amsterdam, The Netherlands

^10^ Section Sports Medicine, Faculty of Health Sciences, University of Pretoria, Pretoria, South Africa

^11^ Football Players Worldwide (FIFPRO), Hoofddorp, The Netherlands

^12^ Centre for Public Health, Queen’s University Belfast, UK

## Expertise:

### Table 1: Demographics of the Project Steering Committee

| **Surname** | **First Name** | **Sex** | **Country of Origin** | **Professional Background** | **Current Professional Role** | **Expertise related to this project** |
| --- | --- | --- | --- | --- | --- | --- |
| Andersen | Thor Einar | Male | Norway | Medical Doctor & PMR Specialist | Professor  & Chief Medical Officer | PhD  Former professional football player. Published >10 papers on heading and head impacts in football. Published Delphi studies across sports-related topics |
| Clarke | Mike | Male | Ireland | Health services research | Professor | DPhil  Published multiple Delphi and consensus studies across a range of health-related topics |
| Dahlen | Sara | Female | Sweden | Medical Doctor | PhD Candidate | Conducting PhD on heading & head impacts in women’s football. |
| Georgieva | Julia | Female | Australia | Physiotherapist | PhD Candidate | Conducting PhD on heading in women’s football with a focus on video coding.  Published 8 studies on heading and/or head injuries in football. |
| Gouttebarge | Vincent | Male | France | Sports and Exercise Medicine Scientist | Chief Medical Officer / Professor | PhD  Former professional footballer |
| Meyer | Tim | Male | Germany | Medical Doctor, Sport Scientist | Full Professor | PhD  Completion of heading research, development of heading guidelines at national and international level |
| Peek* | Kerry | Female | England | Physiotherapist | FIFA Medical Researcher- brain health | PhD  Lead- FIFA Heading Expert Group.  Published 25 studies on heading and/or head injuries in football. |
| Ross | Andrew | Male | Scotland | Physiotherapist | Lecturer and Researcher | PhD  REDCap expertise  Football coaching experience |
| Serner | Andreas | Male | Denmark | Physiotherapist, Clinical Researcher | FIFA Medical Research Lead | PhD  Published 6 studies on heading and/or head injuries in football, and several consensus studies. |
| Williamson | Paula | Female | England | Medical Statistician | Professor | PhD  Published multiple Delphi and consensus studies |

*Chair of Steering Committee

Respondents who agreed to be named and acknowledged for their participation.

*(NB participants indicated in the questionnaire how they were like to be referred as well as self-reporting their role in football):*

### Coaches (18 out of 26):

- Mphatso Chihami
- Bello-Lasoro Abubakar Ola
- Bo Folke Gustavson
- Damir Dugandzic
- Markus Hirte
- Reuben Dauti
- Thom Kazembe
- Michal Ravitz Lurie
- Ellen Chiwenga
- Bongani Mafu
- Stephen Sefolosha
- Terje Julusmoen
- Jani Sarajärvi
- Gijs Luirink
- Precious Oatile
- Edward Ferguson
- Jan Otto Solberg
- Knut Erik Johansen

### Medical Personnel (58 out of 67):

- JL Tol
- Amitesh Kumar
- Joar Harøy
- Guus Reurink
- Gofha Molatedi
- S Veith
- Daniel Adam
- Haruo Nakayama
- Mark L Fulcher
- Benjamin R Kacheuka
- Sunday Olapade
- Mr Munashe Chinyama
- Marc Rizzardo
- M Sahadevan
- George Chiampas
- Dr Charlotte Cowie
- Katherine Forch
- Margot Putukian MD FACSM, FAMSSM
- Cathy Wong
- H Ahmed
- Tendai Moyana
- Lars Engebretsen
- P C A M Lodewijks
- Romain Terrat
- DENIES MAJA
- S. Indeherberge
- Kim Salte
- Ft. Vitor Pimenta
- Ross S Cairns
- Dr Shafaishuna Chase Bassingthwaighte
- Ewen Bradbery
- Dr. Simon Kim
- Dr Mark Jones
- C.A.C.M.Wijne
- Matt Whalan
- Per Olav Kvalvåg
- Johnson KOUAME
- Mustapha Kolawole PT
- Edwin Goedhart
- Joshua M. Smith
- Eric Arosemena
- FT. VITOR PIMENTA
- Ola Sand
- Dr Lisobine H. Kisongo - MD,MMD
- Christopher Rusling
- Kristof Sas
- Thomas T. Ødegaard
- Eva K. Birkelund
- TANAKA ALPHONCE DHIMBIRI
- E Van den Steen
- Brandi Cole
- dr Margaret Maulana
- Alyse F M Cameron
- Malita Kasongo-Phiri
- Kyasiimire Darlian
- Catarina Garcia
- K.N Matavire
- Mohamed Abou Elela A.

### Players (17 out of 33):

- Rick Ketting
- Niek Vossebelt
- Timi Lahti
- Eero-Matti Auvinen
- Mikko Pitkänen
- WK
- Benjamin van den Broek
- Matthew Pennington
- Moshiko Mishaelof
- Albin Granlund
- Ab Persijn
- Mark-Jan Fledderus
- Tori Huster
- Viivi Spets
- Amy Turner
- Alex Culvin
- Rachel Furness

### Researchers (37 out of 41)

- Anthony P. Kontos
- James P Dickey
- Declan A Patton
- Joel D Stitzel
- Jaclyn B. Caccese
- Sara PD Chrisman MD MPH
- Kristy B. Arbogast PhD
- Abigail C Bretzin
- N Virji-Babul
- Kathryn J Schneider
- Nils F H Moseid
- Stian Kirkerud Sandmo
- Stefania Orrù
- Thomas W. Kaminski
- Jillian E Urban
- A Harriss
- Ivy Kondowe Chinangwa
- Steve Rowson
- Thomas Buckley
- Frank Webbe
- Thomas G. Bowman
- Michael L Lipton
- Helene Cassoudesalle
- Gregory Tierney
- John Bjørneboe
- Oliver Faude
- J Evans
- Johannes Weber
- Gregory Dupont
- Barry Drust
- Claus Reinsberger
- José M. Oliva Lozano
- Dr. Florian Beaudouin (PhD)
- Jan Kern
- Tanner M. Filben
- Hege Grindem
- Lyndia Wu

# List of studies used to inform heading descriptors:

*(Published Systematic Review: Peek K, Ross A, Andersen TE, Meyer T, Dahlen S, Georgieva J, Williamson PR, Clarke M, Serner A. Heading in football: a systematic review of descriptors, definitions, and reporting methods used in heading incidence studies. Science and medicine in football. 2024 Jun 10:1-8.)*

| 1. Althoff, K; Kroiher, J; Hennig, EM. A soccer game analysis of two World Cups: playing behavior between elite female and male soccer players. *Footwear Science*. 2010;2(1):51-56. |
| --- |
| 1. Amitay, N; Zlotnik, Y; Coreanu, T; Zeller, L; Abu-Salameh, I; Novack, V; Ifergane, G. Soccer heading and subclinical neuropsychiatric symptomatology in professional soccer players. *Neurology.* 2020;95(13):E1776-E1783. |
| 1. Andersen TB, Bendiksen M, Pedersen JM, Ørntoft C, Brito J, Jackman SR, et al. Kicking velocity and physical, technical, tactical match performance for U18 female football players—effect of a new ball. *Human Movement Science*. 2012;31:1624–38. |
| 1. Beaudouin F, Gioftsidou A, Larsen MN, Lemmink K, Drust B, Modena R, Espinola JR, Meiu M, Vouillamoz M, Meyer T. The UEFA Heading Study: Heading incidence in children’s and youth football (soccer) in eight European countries. *Scandinavian Journal of Medicine & Science in Sports*. 2020Aug;30(8):1506-17. |
| 1. Bonn, MM; Harriss, AB; Thompson, JWG; Dickey, JP. Performing more than 20 purposeful gameplay headers in a soccer season may alter autonomic function in female youth soccer players. *Research in Sports Medicine.* 2021;29(5):440-448. |
| 1. Brooks, JS; Allison, W; Harriss, A; Bian, KW; Mao, HJ; Dickey, JP. Purposeful heading performed by female youth soccer players leads to strain development in deep brain structures. *Neurotrauma Reports.* 2021;2(1):354-362. |
| 1. Caccese JB, Lamond LC, Buckley TA, Kaminski TW. Reducing purposeful headers from goal kicks and punts may reduce cumulative exposure to head acceleration. *Research in Sports Medicine*. 2016 Oct 1;24(4):407-15. |
| 1. Cassoudesalle, H; Bildet, M; Petit, H; Dehail, P. Head impacts in semi-professional male soccer players: a prospective video analysis over one season of competitive games. *Brain Injury.* 2020;34(12):1685-1690. |
| 1. Cassoudesalle, H; Petit, A; Chanraud, S; Petit, H; Badaut, JM; Sibon, I; Dehail, P. Changes in resting-state functional brain connectivity associated with head impacts over one men's semi-professional soccer season. *Journal of Neuroscience Research.* 2021;99(2):446-454. |
| 1. Catenaccio, E; Caccese, J; Wakschlag, N; Fleysher, R; Kim, N; Kim, M; Buckley, TA; Stewart, WF; Lipton, RB; Kaminski, T; Lipton, ML. Validation and calibration of HeadCount, a self-report measure for quantifying heading exposure in soccer players. *Research in Sports Medicine.* 2016;24(4):416-425. |
| 1. Chrisman SP, Mac Donald CL, Friedman S, Andre J, Rowhani-Rahbar A, Drescher S, Stein E, Holm M, Evans N, Poliakov AV, Ching RP, Schwien CC, Vavilala MS, Rivara FP. Head impact exposure during a weekend youth soccer tournament. *Journal of Child Neurology*. 2016 Jul;31(8):971-8. |
| 1. Chrisman SP, Ebel BE, Stein E, Lowry SJ, Rivara FP. Head impact exposure in youth soccer and variation by age and sex. *Clinical Journal of Sport Medicine*. 2019 Jan 1;29(1):3-10. |
| 1. da Silva, CD; Impellizzeri, FM; Natali, AJ; de Lima, JRP; Bara, MG; Silami-Garcia, E; Marins, JCB. Exercise intensity and technical demands of small-sided games in young Brazilian soccer players: effect of number of players, maturation, and reliability. *Journal of Strength and Conditioning Research.* 2011;25(10):2746-2751. |
| 1. Filben, TM; Pritchard, NS; Hanes-Romano, KE; Miller, LE; Miles, CM; Urban, JE; Stitzel, JD. Comparison of women's collegiate soccer header kinematics by play state, intent, and outcome. *Journal of Biomechanics.* 2021;126():110619. |
| 1. Filben, TM; Pritchard, NS; Miller, LE; Miles, CM; Urban, JE; Stitzel, JD. Header biomechanics in youth and collegiate female soccer. *Journal of Biomechanics.* 2021;128():110782 |
| 1. Filben, TM; Pritchard, NS; Miller, LE; Woods, SK; Hayden, ME; Miles, CM; Urban, JE; Stitzel, JD. Characterization of head impact exposure in women's collegiate soccer. *Journal of Applied Biomechanics.* 2022;38(1):2-11. |
| 1. Forbes CR, Glutting JJ, Kaminski TW. Examining cognitive function in previously concussed interscholastic female soccer players. *Applied Neuropsychology Child*. 2016;5:14–24. |
| 1. Hanlon EM, Bir CA. Real-time head acceleration measurement in girls’ youth soccer. *Medicine & Science in Sports & Exercise.* 2012;44:1102–8. |
| 1. Harriss A, Johnson AM, Walton DM, Dickey JP. Head impact magnitudes that occur from purposeful soccer heading depend on the game scenario and head impact location. *Musculoskeletal Science & Practice*. 2019 Apr;40:53-57. |
| 1. Harriss A, Johnson AM, Walton DM, Dickey JP. The number of purposeful headers female youth soccer players experience during games depends on player age but not player position. *Science and Medicine in Football*. 2019 Apr 3;3(2):109-14. |
| 1. Harriss A, Walton DM, Dickey JP. Direct player observation is needed to accurately quantify heading frequency in youth soccer. *Research in Sports Medicine*. 2018 Apr-Jun;26(2):191-198. |
| 1. Huber, CM; Patton, DA; McDonald, CC; Jain, D; Simms, K; Lallo, VA; Margulies, SS; Master, CL; Arbogast, KB. Sport- and gender-based differences in head impact exposure and mechanism in high school sports. *Orthopaedic Journal of Sports Medicine.* 2021;9(3):2325967120984423 |
| 1. Janda, David H; Bir, Cynthia A; Cheney, Angela L. An evaluation of the cumulative concussive effect of soccer heading in the youth population. *Injury Control and Safety Promotion.* 2002;9(1):25-31. |
| 1. Kaminski, TW; Cousino, ES; Glutting, JJ. Examining the relationship between purposeful heading in soccer and computerized neuropsychological test performance. *Research Quarterly For Exercise And Sport.* 2008;79(2):235-244. |
| 1. Kaminski, TW; Weinstein, S; Wahlquist, VE. A comprehensive prospective examination of purposeful heading in American interscholastic and collegiate soccer players. *Science and Medicine in Football.* 2020;4(2):101-110. |
| 1. Kaminski, TW; Wikstrom, AM; Gutierrez, GM; Glutting, JJ. Purposeful heading during a season does not influence cognitive function or balance in female soccer players. *Journal of Clinical and Experimental Neuropsychology.* 2007;29(7):742-751. |
| 1. Kelly DM, Drust B. The effect of pitch dimensions on heart rate responses and technical demands of small-sided soccer games in elite players. *Journal of Science & Medicine in Sport*. 2009;12:475–9. |
| 1. Kenny, R; Elez, M; Clansey, A; Virji-Babul, N; Wu, LC. Head impact exposure and biomechanics in university varsity women's soccer. *Annals of Biomedical Engineering.* 2022;50(11):1461-1472. |
| 1. Kern, J; Lober, T; Hermsdorfer, J; Endo, S. A neural network for the detection of soccer headers from wearable sensor data. *Scientific Reports.* 2022;12(1):18128 |
| 1. Koerte IK, Nichols E, Tripodis Y, Schultz V, Lehner S, Igbinoba R, et al. Impaired cognitive performance in youth athletes exposed to repetitive head impacts. *Journal of Neurotrauma*. 2017;34:2389–95. |
| 1. Kontos AP, Dolese A, Elbin RJ, Covassin T, Warren BL. Relationship of soccer heading to computerized cognitive performance and symptoms among female and male youth soccer players. *Brain Injury.* 2011;25:1234–41. |
| 1. Lamond, LC; Caccese, JB; Buckley, TA; Glutting, J; Kaminski, TW. Linear Acceleration in direct head contact across impact type, player position, and playing scenario in collegiate women's soccer players. *Journal of Athletic Training*. 2018;53(2):115-121. |
| 1. Langdon, S; Goedhart, E; Oosterlaan, J; Konigs, M. Heading exposure in elite football (soccer): a study in adolescent, young adult, and adult male and female players. *Medicine & Science in Sports & Exercise.* 2022;54(9):1459-1465. |
| 1. Martone, D; Giacobbe, M; Capobianco, A; Imperlini, E; Mancini, A; Capasso, M; Buono, P; Orru, S. Exercise intensity and technical demands of small-sided soccer games for under-12 and under-14 players: effect of area per player. *Journal of Strength and Conditioning Research.* 2017;31(6):1486-1492. |
| 1. Miller, LE; Pinkerton, EK; Fabian, KC; Wu, LC; Espeland, MA; Lamond, LC; Miles, CM; Camarillo, DB; Stitzel, JD; Urban, JE. Characterizing head impact exposure in youth female soccer with a custom-instrumented mouthpiece. *Research in Sports Medicine.* 2020;28(1):55-71. |
| 1. Nevins, D.; Hildenbrand, K.; Vasavada, A.; Kensrud, J.; Smith, L. In-game head impact exposure of male and female high school soccer players. *Athletic Training and Sports Health Care.* 2019;11:174–182. |
| 1. Nevins, D; Hildenbrand, K; Kensrud, J; Vasavada, A; Smith, L. Field evaluation of a small form-factor head impact sensor for use in soccer. *Engineering of Sport*.2016;147:186-190. |
| 1. Nevins, D; Hildenbrand, K; Kensrud, J; Vasavada, A; Smith, L. Laboratory and field evaluation of a small form factor head impact sensor in un-helmeted play. *Proceedings of The Institution of Mechanical Engineers Part P: Journal Of Sports Engineering and Technology*. 2018;232(3):242-254. |
| 1. Owen, AL; Wong, DP; McKenna, M; Dellal, A. Heart rate responses and technical comparison between small- vs. large-sided games in elite professional soccer. *Journal of Strength and Conditioning Research.* 2011;25(8):2104-2110. |
| 1. Patton, DA; Huber, CM; Margulies, SS; Master, CL; Arbogast, KB. Comparison of video-identified head contacts and sensor-recorded events in high school soccer. *Journal of Applied Biomechanics.* 2021;37(6):573-577. |
| 1. Patton, DA; Huber, CM; McDonald, CC; Margulies, SS; Master, CL; Arbogast, KB. Video confirmation of head impact sensor data from high school soccer players. *American Journal of Sports Medicine.* 2020;48(5):1246-1253. |
| 1. Peek K, Meyer T, Beaudouin F, McKay M. Heading incidence in boys’ football over three seasons. *Science and Medicine in Football*. 2021 Jul 3;5(3):175-80. |
| 1. Peek K, Vella T, Meyer T, Beaudouin F, McKay M. The incidence and characteristics of purposeful heading in male and female youth football (soccer) within Australia. *Journal of Science and Medicine in Sport*. 2021 Jun 1;24(6):603-8. |
| 1. Porfido, T; Caccese, J; Gutt, J; Wentworth, C; Peek, K; Bretzin, AC; Esopenko, C.A standardized method for quantifying and characterizing repetitive head impacts in soccer matches using video footage. *Science and Medicine in Football.* 2022;6(3):331-339. |
| 1. Press, JN; Rowson, S. Quantifying head impact exposure in collegiate women's soccer. *Clinical Journal of Sport Medicine*. 2017;27(2):104-110. |
| 1. Pritchard, NS; Filben, TM; Haja, SJ; Miller, LE; Espeland, MA; Stitzel, JD; Urban, JE. Head impact exposure in youth soccer: comparing across activity types. Proceedings of The Institution of Mechanical Engineers Part P: Journal Of Sports Engineering and Technology 2023. |
| 1. Rago V, Rebelo AN, Pizzuto F, Barreira D. Small-sided soccer games on sand are more physically demanding but less technically specific compared to games on artificial turf. *Journal of Sports Medicine and Physical Fitness*. 2018;58:385–91. |
| 1. Rahnama, N; Reilly, T; Lees, A. Injury risk associated with playing actions during competitive soccer. *British Journal of Sports Medicine.* 2002;36(5):354-359. |
| 1. Reeschke, R; Haase, FK; Dautzenberg, L; Krutsch, W; Reinsberger, C. Training matters: Heading incidence and characteristics in children's and youth football (soccer) players. *Scandinavian Journal of Medicine & Science in Sports.* 2023;33(9):1821-1830 |
| 1. Rich, AM; Filben, TM; Miller, LE; Tomblin, BT; Van Gorkom, AR; Hurst, MA; Barnard, RT; Kohn, DS; Urban, JE; Stitzel, JD. Development, validation and pilot field deployment of a custom mouthpiece for head impact measurement. *Annals of Biomedical Engineering.* 2019;47(10):2109-2121. |
| 1. Roman I, McKay M, Peek, K. Head impact events in youth football in India and Australia, compared to FIFA Men's World Cup matches. *Journal of Science and Medicine in Sport Plus*. 2023;2:100029. |
| 1. Rutherford A, Stephens R, Fernie G, Potter D. Do UK university football club players suffer neuropsychological impairment as a consequence of their football (soccer) play? *Journal of Clinical and Experimental Neuropsychology*.2009;31:664–81. |
| 1. Salinas, CM; Webbe, FM; Devore, TT.The epidemiology of soccer heading in competitive youth players. *Journal of Clinical Sport Psychology.2009;3(1):15-33.* |
| 1. Sandmo SB, Andersen TE, Koerte IK, Bahr R. Head impact exposure in youth football—Are current interventions hitting the target?. *Scandinavian Journal of Medicine & Science in Sports*. 2020 Jan;30(1):193-8. |
| 1. Sandmo, SB; Filipcik, P; Cente, M; Hanes, J; Andersen, TE; Straume-Naesheim, TM; Bahr, R. Neurofilament light and tau in serum after head-impact exposure in soccer. *Brain Injury.* 2020;34(5):602-609. |
| 1. Sandmo, SB; Gooijers, J; Seer, C; Kaufmann, D; Bahr, R; Pasternak, O; Lipton, ML; Tripodis, Y; Koerte, IK. Evaluating the validity of self-report as a method for quantifying heading exposure in male youth soccer. *Research in Sports Medicine.* 2021;29(5):427-439. |
| 1. Sandmo, SB; Mcintosh, AS; Andersen, TE; Koerte, IK; Bahr, R. Evaluation of an in-ear sensor for quantifying head impacts in youth soccer. *American Journal of Sports Medicine.* 2019;47(4):974-981. |
| 1. Sarajarvi, J; Volossovitch, A; Almeida, CH. Analysis of headers in high-performance football: evidence from the English Premier League. *International Journal of Performance Analysis in Sport.* 2020;20(2):189-205. |
| 1. Sattari, S; Kenny, R; Liu, CC; Hajra, SG; Dumont, GA; Virji-Babul, N. Blink-related EEG oscillations are neurophysiological indicators of subconcussive head impacts in female soccer players: a preliminary study. *Frontiers in Human Neuroscience.* 2023;17(:1208498 |
| 1. Saunders, TD; Le, RK; Breedlove, KM; Bradney, DA; Bowman, TG. Sex differences in mechanisms of head impacts in collegiate soccer athletes. *Clinical Biomechanics.* 2020;74():14-20. |
| 1. Segars, MF; Filben, TM; Pritchard, NS; Miller, LE; Miles, CM; Stitzel, JD; Urban, JE. Head impact exposure in female collegiate soccer by activity type. *Journal of Applied Biomechanics.* 2023;39(4):209-216. |
| 1. Sokol-Randell, D; Stelzer-Hiller, OW; Allan, D; Tierney, G. Heads up! A biomechanical pilot investigation of soccer heading using instrumented mouthguards (iMGs).*Applied Sciences-Basel.* 2023;13(4):2639 |
| 1. Stalnacke, BM; Ohlsson, A; Tegner, Y; Sojka, P. Serum concentrations of two biochemical markers of brain tissue damage S-100B and neurone specific enolase are increased in elite female soccer players after a competitive game. *British Journal of Sports Medicine.* 2006;40(4):313-316. |
| 1. Stalnacke, BM; Tegner, Y; Sojka, P. Playing soccer increases serum concentrations of the biochemical markers of brain damage S-100B and neuron-specific enolase in elite players: a pilot study. *Brain Injury.* 2004;18(9):899-909. |
| 1. Stephens R, Rutherford A, Potter D, Fernie G. Neuropsychological consequence of soccer play in adolescent U.K. school team soccer players. *Journal of Neuropsychiatry and Clinical Neurosciences*. 2010;22:295–303. |
| 1. Straume-Naesheim, TM; Andersen, TE; Dvorak, J; Bahr, R. Effects of heading exposure and previous concussions on neuropsychological performance among Norwegian elite footballers. *British Journal of Sports Medicine.* 2005;39:I70-I77. |
| 1. Tierney, GJ; Higgins, B. The incidence and mechanism of heading in European professional football players over three seasons. *Scandinavian Journal of Medicine & Science in Sports.* 2021;31(4):875-883. |
| 1. Tomblin, BT; Pritchard, NS; Filben, TM; Miller, LE; Miles, CM; Urban, JE; Stitzel, JD. Characterization of on-field head impact exposure in youth soccer. *Journal of Applied Biomechanics.* 2021;37(1):36-42. |
| 1. Uebersax, J; Roth, R; Bachle, T; Faude, O. Structure, intensity and player duels in under-13 football training in Switzerland. *International Journal of Environmental Research and Public Health.* 2020;17(22):1-13. |
| 1. Weber J, Ernstberger A, Reinsberger C, Popp D, Nerlich M, Alt V, et al. Video analysis of 100 matches in male semi-professional football reveals a heading rate of 5.7 headings per field player and match. *BMC Sports Science, Medicine & Rehabilitation*. 2022;14(1):1-132. |
| 1. Weber, J; Reinsberger, C; Krutsch, V; Seiffert, R; Huber, L; Alt, V; Krutsch, W. Heading and risk of injury situations for the head in professional German football: a video analysis of over 150,000 headers in 110,000 match minutes. *Science And Medicine in Football.* 2022;(ahead-of-print):1-8. |

# Invitees including responders and non-responders

Table 2 includes the demographics of people invited to participate in this project, including those who responded, and those who did not. Missing data for age, gender, and age group of players that the person works with exists for non-responders as they did not complete the demographic questions included I the first questionnaire. Players also did not complete questions in the last two columns. In addition, 17 emails were returned as undeliverable. All from researchers.

### Table 2: Invitees including responders and non-responders

| **Responded to at least one survey** | **Role initially assigned** | **Role-**  **self report** | **Age** | **Gender** | **Country** | **Confederation** | **Gender of players mainly work with** | **Age group of players mainly work with** |
| --- | --- | --- | --- | --- | --- | --- | --- | --- |
| Yes | Coach | Coach | 59 | Man | Norway | UEFA | Men/ boys | Adolescents (13-17 years) |
| Yes | Coach | Coach | 58 | Man | Zimbabwe | UEFA | Men/ boys | Adults (18 years and older) |
| Yes | Coach | Coach | 56 | Man | Norway | UEFA | Men/ boys | Children (12 years or younger) |
| Yes | Coach | Coach | 23 | Man | Malawi | CAF | Fairly even split between Men/ boys and Women/ girls | Adolescents (13-17 years) |
| Yes | Coach | Coach | 48 | Man | Germany | UEFA | Men/ boys | Adolescents (13-17 years) |
| Yes | Coach | Coach | 48 | Man | Botswana | CAF | Men/ boys | Adults (18 years and older) |
| Yes | Coach | Coach | 51 | Woman | Zimbabwe | CAF | Fairly even split between Men/ boys and Women/ girls | Adults (18 years and older) |
| Yes | Coach | Researcher | 24 | Man | England | UEFA | Men/ boys | Adults (18 years and older) |
| Yes | Coach | Coach | 50 | Man | Netherlands | UEFA | Men/ boys | Adults (18 years and older) |
| Yes | Coach | Coach |  | Man | Norway | UEFA | Fairly even split between Men/ boys and Women/ girls | Adolescents (13-17 years) |
| Yes | Coach | Coach | 55 | Man | Norway | UEFA | Men/ boys | Adults (18 years and older) |
| Yes | Coach | Coach | 28 | Woman | Botswana | CAF | Men/ boys | Adults (18 years and older) |
| Yes | Coach | Coach | 37 | Woman | Israel | UEFA | Women/ girls | Adolescents (13-17 years) |
| Yes | Coach | Coach | 42 | Man | Zimbabwe | CAF | Men/ boys | Adults (18 years and older) |
| Yes | Coach | Coach | 31 | Man | Australia | AFC | Fairly even split between Men/ boys and Women/ girls | Children (12 years or younger) |
| Yes | Coach | Medical personnel | 43 | Man | Netherlands | UEFA | Fairly even split between Men/ boys and Women/ girls | Adults (18 years and older) |
| Yes | Coach | Coach | 40 | Man | Netherlands | UEFA | Men/ boys | Adults (18 years and older) |
| Yes | Coach | Coach | 47 | Man | Norway | UEFA | Men/ boys | Adults (18 years and older) |
| Yes | Coach | Player | 38 | Man | Zimbabwe | CAF | Men/ boys | Adolescents (13-17 years) |
| Yes | Coach | Coach | 39 | Woman | Zambia | CAF | Women/ girls | Adolescents (13-17 years) |
| Yes | Coach | Coach | 61 | Man | Germany | UEFA | Men/ boys | Adolescents (13-17 years) |
| Yes | Coach | Medical personnel | 38 | Man | South Africa | CAF | Men/ boys | Adults (18 years and older) |
| Yes | Coach | Coach | 46 | Man | Malawi | CAF | Women/ girls | Adolescents (13-17 years) |
| Yes | Coach | Player | 32 | Woman | England | UEFA | Women/ girls | Adults (18 years and older) |
| Yes | Coach | Coach | 34 | Man | Nigeria | CAF | Fairly even split between Men/ boys and Women/ girls | Adolescents (13-17 years) |
| Yes | Coach | Player | 32 | Woman | England | UEFA | Women/ girls | Adults (18 years and older) |
| Yes | Coach | Coach | 54 | Man | Norway | UEFA | Men/ boys | Children (12 years or younger) |
| Yes | Coach | Coach | 40 | Man | Malawi | CAF | Women/ girls | Adolescents (13-17 years) |
| Yes | Coach | Researcher |  | Man | England | UEFA | Men/ boys | Adults (18 years and older) |
| Yes | Coach | Researcher |  | Man | Spain | UEFA | Men/ boys | Adults (18 years and older) |
| Yes | Coach | Researcher | 52 | Woman | Malawi | CAF | Women/ girls | Adolescents (13-17 years) |
| Yes | Coach | Coach |  | Woman | Zimbabwe | CAF | Women/ girls | Adults (18 years and older) |
| Yes | Coach | coach | 33 | Woman | Zambia | CAF | Fairly even split between Men/ boys and Women/ girls | Adolescents (13-17 years) |
| No | Coach |  |  | Man | Zimbabwe | CAF |  |  |
| No | Coach |  |  | Man | Netherlands | UEFA |  |  |
| No | Coach |  |  | Man | Botswana | CAF |  |  |
| No | Coach |  |  | Man | England | UEFA |  |  |
| No | Coach |  |  | Man | England | UEFA |  |  |
| No | Coach |  |  | Man | Ireland | UEFA |  |  |
| No | Coach |  |  | Man | Netherlands | UEFA |  |  |
| No | Coach |  |  | Woman | Netherlands | UEFA |  |  |
| No | Coach |  |  | Man | Zimbabwe | CAF |  |  |
| No | Coach |  |  | Man | Norway | UEFA |  |  |
| No | Coach |  |  | Woman | Norway | UEFA |  |  |
| No | Coach |  |  | Man | Norway | UEFA |  |  |
| No | Coach |  |  | Man | Malawi | CAF |  |  |
| No | Coach |  |  | Woman | South Africa | CAF |  |  |
| No | Coach |  |  | Man | Zimbabwe | CAF |  |  |
| No | Coach |  |  | Man | Norway | UEFA |  |  |
| No | Coach |  |  | Man | Norway | UEFA |  |  |
| No | Coach |  |  | Man | Zimbabwe | CAF |  |  |
| No | Coach |  |  | Man | Norway | UEFA |  |  |
| No | Coach |  |  | Woman | Norway | UEFA |  |  |
| No | Coach |  |  | Man | Zimbabwe | CAF |  |  |
| No | Coach |  |  | Man | Uganda | CAF |  |  |
| No | Coach |  |  | Man | Zimbabwe | CAF |  |  |
| No | Coach |  |  | Man | Norway | UEFA |  |  |
| No | Coach |  |  | Man | Zimbabwe | CAF |  |  |
| Yes | Medical | Medical personnel | 43 | Man | England | UEFA | Men/ boys | Adults (18 years and older) |
| Yes | Medical | Medical personnel | 25 | Man | Malawi | CAF | Fairly even split between Men/ boys and Women/ girls | Adults (18 years and older) |
| Yes | Medical | Medical personnel | 55 | Man | Netherlands | UEFA | Fairly even split between Men/ boys and Women/ girls | Adolescents (13-17 years) |
| Yes | Medical | Medical personnel | 36 | Man | Zimbabwe | CAF | Men/ boys | Adults (18 years and older) |
| Yes | Medical | Medical personnel | 37 | Man | Netherlands | UEFA | Men/ boys | Adults (18 years and older) |
| Yes | Medical | Medical personnel | 59 | Woman | England | UEFA | Fairly even split between Men/ boys and Women/ girls | Adults (18 years and older) |
| Yes | Medical | Medical personnel | 47 | Man | Japan | AFC | Fairly even split between Men/ boys and Women/ girls | Adults (18 years and older) |
| Yes | Medical | Medical personnel | 42 | Man | Norway | UEFA | Men/ boys | Adolescents (13-17 years) |
| Yes | Medical | Medical personnel | 33 | Man | New Caledonia | OFC | Men/ boys | Adults (18 years and older) |
| Yes | Medical | Medical personnel | 47 | Man | New Zealand | OFC | Fairly even split between Men/ boys and Women/ girls | Adults (18 years and older) |
| Yes | Medical | Medical personnel | 29 | Man | Republic of South Africa | CAF | Men/ boys | Adults (18 years and older) |
| Yes | Medical | Medical personnel | 45 | Man | Belgium | UEFA | Men/ boys | Adults (18 years and older) |
| Yes | Medical | Researcher | 53 | Man | Norway | UEFA | Men/ boys | Adolescents (13-17 years) |
| Yes | Medical | Medical personnel | 42 | Woman | Belgium | UEFA | Women/ girls | Adults (18 years and older) |
| Yes | Medical | Medical personnel | 25 | Woman | Zimbabwe | CAF | Men/ boys | Adults (18 years and older) |
| Yes | Medical | Medical personnel | 40 | Man | Fiji | OFC | Fairly even split between Men/ boys and Women/ girls | Adolescents (13-17 years) |
| Yes | Medical | Medical personnel | 28 | Man | Malawi | CAF | Fairly even split between Men/ boys and Women/ girls | Adults (18 years and older) |
| Yes | Medical | Medical personnel | 36 | Man | Norway | UEFA | Men/ boys | Adults (18 years and older) |
| Yes | Medical | Medical personnel | 27 | Man | Malawi | CAF | Fairly even split between Men/ boys and Women/ girls | Adults (18 years and older) |
| Yes | Medical | Medical personnel | 51 | Man | Portugal | UEFA | Men/ boys | Adults (18 years and older) |
| Yes | Medical | Medical personnel | 43 | Man | Australia | AFC | Men/ boys | Adults (18 years and older) |
| Yes | Medical | Medical personnel | 41 | Woman | Australia | AFC | Women/ girls | Adults (18 years and older) |
| Yes | Medical | Medical personnel | 34 | Man | Namibia | CAF | Men/ boys | Adults (18 years and older) |
| Yes | Medical | Medical personnel | 36 | Woman | New Zealand | OFC | Fairly even split between Men/ boys and Women/ girls | Adults (18 years and older) |
| Yes | Medical | Medical personnel | 33 | Man | Australia | AFC | Men/ boys | Adults (18 years and older) |
| Yes | Medical | Medical personnel | 49 | Man | England | UEFA | Men/ boys | Adults (18 years and older) |
| Yes | Medical | Medical personnel | 25 | Man | Zimbabwe | CAF | Women/ girls | Adults (18 years and older) |
| Yes | Medical | Medical personnel | 42 | Man | New Zealand | OFC | Men/ boys | Adults (18 years and older) |
| Yes | Medical | Medical personnel | 63 | Man | Australia | AFC | Men/ boys | Adults (18 years and older) |
| Yes | Medical | Medical personnel | 57 | Man | Netherlands | UEFA | Men/ boys | Adults (18 years and older) |
| Yes | Medical | Medical personnel | 45 | Woman | Norway | UEFA | Fairly even split between Men/ boys and Women/ girls | Adults (18 years and older) |
| Yes | Medical | Medical personnel | 44 | Man | Australia | AFC | Men/ boys | Adults (18 years and older) |
| Yes | Medical | Medical personnel | 42 | Man | Norway | UEFA | Men/ boys | Adults (18 years and older) |
| Yes | Medical | Medical personnel | 39 | Man | Nigeria | CAF | Fairly even split between Men/ boys and Women/ girls | Adults (18 years and older) |
| Yes | Medical | Medical personnel | 38 | Man | Côte d'Ivoire | CAF | Men/ boys | Adults (18 years and older) |
| Yes | Medical | Medical personnel | 53 | Woman | Norway | UEFA | Men/ boys | Adults (18 years and older) |
| Yes | Medical | Medical personnel | 57 | Woman | Zimbabwe | CAF | Women/ girls | Adults (18 years and older) |
| Yes | Medical | Medical personnel | 44 | Man | Nigeria | CAF | Men/ boys | Adults (18 years and older) |
| Yes | Medical | Medical personnel | 38 | Man | Egypt | CAF | Fairly even split between Men/ boys and Women/ girls | Adults (18 years and older) |
| Yes | Medical | Medical personnel | 32 | Woman | Australia | AFC | Women/ girls | Adolescents (13-17 years) |
| Yes | Medical | Medical personnel | 60 | Man | Netherlands | UEFA | Men/ boys | Adults (18 years and older) |
| Yes | Medical | Medical personnel | 41 | Man | Netherlands | UEFA | Men/ boys | Adolescents (13-17 years) |
| Yes | Medical | Medical personnel | 38 | Woman | New Zealand | OFC | Women/ girls | Adults (18 years and older) |
| Yes | Medical | Medical personnel | 36 | Woman | Zambia | CAF | Women/ girls | Adults (18 years and older) |
| Yes | Medical | Medical personnel | 39 | Man | Botswana | CAF | Men/ boys | Adolescents (13-17 years) |
| Yes | Medical | Medical personnel | 38 | Woman | New Zealand | OFC | Women/ girls | Adults (18 years and older) |
| Yes | Medical | Medical personnel | 27 | Woman | Uganda | CAF | Women/ girls | Adults (18 years and older) |
| Yes | Medical | Medical personnel | 39 | Man | Panama | CONCACAF | Men/ boys | Adults (18 years and older) |
| Yes | Medical | Medical personnel | 61 | Woman | United States of America | CONCACAF | Fairly even split between Men/ boys and Women/ girls | Adults (18 years and older) |
| Yes | Medical | Medical personnel | 60 | Woman | Fiji | OFC | Fairly even split between Men/ boys and Women/ girls | Adults (18 years and older) |
| Yes | Medical | Medical personnel | 39 | Woman | Brazil | CONMEBOL | Women/ girls | Adults (18 years and older) |
| Yes | Medical | Medical personnel | 30 | Woman | Zambia | CAF | Women/ girls | Adults (18 years and older) |
| Yes | Medical | Medical personnel | 32 | Man | Namibia | CAF | Fairly even split between Men/ boys and Women/ girls | Adults (18 years and older) |
| Yes | Medical | Medical personnel | 51 | Man | Portugal | UEFA | Men/ boys | Adults (18 years and older) |
| Yes | Medical | Medical personnel | 68 | Man | Norway | UEFA | Men/ boys | Adults (18 years and older) |
| Yes | Medical | Medical personnel | 68 | Man | Canada | CONCACAF | Fairly even split between Men/ boys and Women/ girls | Adults (18 years and older) |
| Yes | Medical | Medical personnel | 52 | Man | Tanzania | CAF | Men/ boys | Adults (18 years and older) |
| Yes | Medical | Medical personnel | 34 | Man | Canada | CONCACAF | Men/ boys | Adults (18 years and older) |
| Yes | Medical | Medical personnel | 45 | Man | Malaysia | AFC | Fairly even split between Men/ boys and Women/ girls | Adults (18 years and older) |
| Yes | Medical | Medical personnel | 48 | Man | Belgium | UEFA | Men/ boys | Adults (18 years and older) |
| Yes | Medical | Researcher | 50 | Woman | Canada | CONCACAF | Fairly even split between Men/ boys and Women/ girls | Adolescents (13-17 years) |
| Yes | Medical | Medical personnel | 53 | Man | Norway | UEFA | Men/ boys | Adults (18 years and older) |
| Yes | Medical | Medical personnel | 52 | Man | United states | CONCACAF | Fairly even split between Men/ boys and Women/ girls | Adults (18 years and older) |
| Yes | Medical | Medical personnel | 27 | Woman | Zimbabwe | CAF | Women/ girls | Adults (18 years and older) |
| No | Medical |  |  | Woman | Zimbabwe | UEFA |  |  |
| No | Medical |  |  | Man | Ireland | UEFA |  |  |
| No | Medical |  |  | Man | Cameron | CAF |  |  |
| No | Medical |  |  | Man | Panama | CONCACAF |  |  |
| No | Medical |  |  | Woman | Panama | CONCACAF |  |  |
| No | Medical |  |  | Woman | Belgium | UEFA |  |  |
| No | Medical |  |  | Man | Norway | UEFA |  |  |
| No | Medical |  |  | Man | Bermuda | CONCACAF |  |  |
| No | Medical |  |  | Man | New Caledonia | OFC |  |  |
| No | Medical |  |  | Man | Norway | UEFA |  |  |
| No | Medical |  |  | Woman | Jamaica | CONCACAF |  |  |
| No | Medical |  |  | Woman | Eswatini | CAF |  |  |
| No | Medical |  |  | Woman | Netherlands | UEFA |  |  |
| No | Medical |  |  | Man | Nigeria | CAF |  |  |
| No | Medical |  |  | Man | Ghana | CAF |  |  |
| Yes | Player | Player | 24 | Woman | Finland | UEFA |  |  |
| Yes | Player | Player | 30 | Woman | United states | CONCACAF |  |  |
| Yes | Player | Player | 28 | Man | Netherlands | UEFA |  |  |
| Yes | Player | Player | 27 | Woman | United states | CONCACAF |  |  |
| Yes | Player | Player | 34 | Woman | United states | CONCACAF |  |  |
| Yes | Player | Player | 17 | Woman | Israel | UEFA |  |  |
| Yes | Player | Player | 25 | Woman | Finland | UEFA |  |  |
| Yes | Player | Player | 32 | Man | Netherlands | UEFA |  |  |
| Yes | Player | Player | 33 | Man | Finland | UEFA |  |  |
| Yes | Player | Player | 28 | Man | Finland | UEFA |  |  |
| Yes | Player | Player | 22 | Woman | Finland | UEFA |  |  |
| Yes | Player | Player | 27 | Woman | Finland | UEFA |  |  |
| Yes | Player | Player | 27 | Man | Finland | UEFA |  |  |
| Yes | Player | Player | 36 | Man | Netherlands | UEFA |  |  |
| Yes | Player | Player | 29 | Man | England | UEFA |  |  |
| Yes | Player | Player | 35 | Man | Ireland | UEFA |  |  |
| Yes | Player | Player | 21 | Woman | Finland | UEFA |  |  |
| Yes | Player | Player | 39 | Woman | Sweden | UEFA |  |  |
| Yes | Player | Player | 25 | Woman | United states | CONCACAF |  |  |
| Yes | Player | Player | 40 | Woman | United states | CONCACAF |  |  |
| Yes | Player | Player | 49 | Man | Norway | UEFA |  |  |
| Yes | Player | Player | 40 | Man | Israel | UEFA |  |  |
| Yes | Player | Player | 20 | Woman | Nederland | UEFA |  |  |
| Yes | Player | Player |  | Man | Finland | UEFA |  |  |
| Yes | Player | Player | 31 | Woman | Netherlands | UEFA |  |  |
| Yes | Player | Player | 40 | Woman | England | UEFA |  |  |
| Yes | Player | Player | 35 | Woman | England | UEFA |  |  |
| Yes | Player | Player | 50 | Man | Netherlands | UEFA |  |  |
| Yes | Player | Player | 41 | Man | Netherlands | UEFA |  |  |
| No | Player |  |  | Man | Israel | UEFA |  |  |
| No | Player |  |  | Man | Netherlands | UEFA |  |  |
| No | Player |  |  | Man | Israel | UEFA |  |  |
| No | Player |  |  | Man | Finland | UEFA |  |  |
| No | Player |  |  | Man | Israel | UEFA |  |  |
| No | Player |  |  | Woman | Israel | UEFA |  |  |
| No | Player |  |  | Man | Netherlands | UEFA |  |  |
| No | Player |  |  | Man | Netherlands | UEFA |  |  |
| No | Player |  |  | Man | Israel | UEFA |  |  |
| No | Player |  |  | Man | Netherlands | UEFA |  |  |
| No | Player |  |  | Man | Israel | UEFA |  |  |
| No | Player |  |  | Man | England | UEFA |  |  |
| Yes | Researcher | Medical personnel | 74 | Man | Norway | UEFA | Men/ boys | Adults (18 years and older) |
| Yes | Researcher | Researcher | 33 | Woman | United states | CONCACAF | Fairly even split between Men/ boys and Women/ girls | Adolescents (13-17 years) |
| Yes | Researcher | Researcher | 40 | Man | United states | CONCACAF | Fairly even split between Men/ boys and Women/ girls | Adults (18 years and older) |
| Yes | Researcher | Researcher | 31 | Man | Ireland | UEFA | Men/ boys | Adults (18 years and older) |
| Yes | Researcher | Researcher | 52 | Woman | United states | CONCACAF | Fairly even split between Men/ boys and Women/ girls | Adolescents (13-17 years) |
| Yes | Researcher | Researcher | 39 | Man | Norway | UEFA | Men/ boys | Adults (18 years and older) |
| Yes | Researcher | Researcher | 54 | Man | United states | CONCACAF | Fairly even split between Men/ boys and Women/ girls | Adolescents (13-17 years) |
| Yes | Researcher | Researcher | 32 | Man | Germany | UEFA | Women/ girls | Adults (18 years and older) |
| Yes | Researcher | Player | 44 | Man | Germany | UEFA | Fairly even split between Men/ boys and Women/ girls | Adults (18 years and older) |
| Yes | Researcher | Researcher | 53 | Woman | United states | CONCACAF | Fairly even split between Men/ boys and Women/ girls | Adolescents (13-17 years) |
| Yes | Researcher | Researcher | 50 | Man | Germany | UEFA | Men/ boys | Adults (18 years and older) |
| Yes | Researcher | Researcher | 62 | Man | United states | CONCACAF | Women/ girls | Adolescents (13-17 years) |
| Yes | Researcher | Researcher | 50 | Man | United states | CONCACAF | Fairly even split between Men/ boys and Women/ girls | Adults (18 years and older) |
| Yes | Researcher | Medical personnel | 66 | Man | Norway | UEFA | Fairly even split between Men/ boys and Women/ girls | Adolescents (13-17 years) |
| Yes | Researcher | Researcher | 40 | Woman | Norway | UEFA | Women/ girls | Adults (18 years and older) |
| Yes | Researcher | Researcher | 76 | Man | United states | CONCACAF | Fairly even split between Men/ boys and Women/ girls | Adults (18 years and older) |
| Yes | Researcher | Researcher | 35 | Woman | Canada | CONCACAF | Women/ girls | Adults (18 years and older) |
| Yes | Researcher | Researcher | 35 | Man | Norway | UEFA | Men/ boys | Adolescents (13-17 years) |
| Yes | Researcher | Researcher | 61 | Man | Canada | CONCACAF | Fairly even split between Men/ boys and Women/ girls | Adolescents (13-17 years) |
| Yes | Researcher | Researcher | 37 | Man | Germany | UEFA | Men/ boys | Adults (18 years and older) |
| Yes | Researcher | Medical personnel |  | Man | Norway | UEFA | Men/ boys | Adults (18 years and older) |
| Yes | Researcher | Researcher | 36 | Woman | United states | CONCACAF | Women/ girls | Adolescents (13-17 years) |
| Yes | Researcher | Researcher | 32 | Woman | United states | CONCACAF | Fairly even split between Men/ boys and Women/ girls | Adolescents (13-17 years) |
| Yes | Researcher | Coach | 44 | Man | Finland | UEFA | Men/ boys | Adults (18 years and older) |
| Yes | Researcher | Researcher | 43 | Man | United states | CONCACAF | Fairly even split between Men/ boys and Women/ girls | Adults (18 years and older) |
| Yes | Researcher | Researcher | 35 | Man | Norway | UEFA | Men/ boys | Adolescents (13-17 years) |
| Yes | Researcher | Researcher | 51 | Man | France | UEFA | Men/ boys | Adults (18 years and older) |
| Yes | Researcher | Researcher | 43 | Man | Brazil | CONMEBOL | Men/ boys | Adults (18 years and older) |
| Yes | Researcher | Researcher |  | Prefer not to state | United states | CONCACAF | Fairly even split between Men/ boys and Women/ girls | Adolescents (13-17 years) |
| Yes | Researcher | Researcher | 38 | Woman | France | UEFA | Fairly even split between Men/ boys and Women/ girls | Adults (18 years and older) |
| Yes | Researcher | Researcher | 54 | Man | England | UEFA | Men/ boys | Adults (18 years and older) |
| Yes | Researcher | Researcher | 58 | Man | United states | CONCACAF | Fairly even split between Men/ boys and Women/ girls | Adults (18 years and older) |
| Yes | Researcher | Researcher | 49 | Man | Germany | UEFA | Men/ boys | Adults (18 years and older) |
| Yes | Researcher | Coach | 39 | Man | Zimbabwe | CAF | Men/ boys | Adolescents (13-17 years) |
| Yes | Researcher | Researcher | 54 | Woman | Italy | UEFA | Men/ boys | Adolescents (13-17 years) |
| Yes | Researcher | Researcher | 39 | Man | United states | CONCACAF | Fairly even split between Men/ boys and Women/ girls | Adolescents (13-17 years) |
| Yes | Researcher | Researcher | 28 | Man | United states | CONCACAF | Women/ girls | Adults (18 years and older) |
| Yes | Researcher | Researcher |  | Woman | Canada | CONCACAF | Fairly even split between Men/ boys and Women/ girls | Adolescents (13-17 years) |
| Yes | Researcher | Researcher | 33 | Woman | Canada | CONCACAF | Women/ girls | Adolescents (13-17 years) |
| Yes | Researcher | Researcher | 36 | Man | Germany | UEFA | Men/ boys | Adults (18 years and older) |
| Yes | Researcher | Researcher | 28 | Man | United states | CONCACAF | Women/ girls | Adolescents (13-17 years) |
| No | Researcher |  |  | Man | Portugal | UEFA |  |  |
| No | Researcher |  |  | Man | Norway | UEFA |  |  |
| No | Researcher |  |  | Woman | Netherlands | UEFA |  |  |
| No | Researcher |  |  | Man | Denmark | UEFA |  |  |
| No | Researcher |  |  | Man | Israel | UEFA |  |  |
| No | Researcher |  |  | Man | United states | CONCACAF |  |  |
| No | Researcher |  |  | Woman | Germany | UEFA |  |  |
| No | Researcher |  |  | Woman | United states | CONCACAF |  |  |

# Questionnaires

This project included five questionnaires:

Phase 1

- First round questionnaire
- Second round questionnaire

Phase 2

- First round questionnaire
- Second round questionnaire

Phase 3

- First round questionnaire

# Results

## Phase 1- First Round Questionnaire

Phase 1 included two questionnaires, round-one and round-two. These questionnaires focused on gaining >80% consensus on the descriptors that should be included in minimum reporting criteria for heading incidence research.

Table 3 contains the results from the Phase 1- first round questionnaire for all participants and per role.

*Key:*

*Green: >80% to include*

*Pink: >80% to exclude*

### Table 3: Results for Phase 1- first-round questionnaire for all participants and per role (%)

| **Descriptor** | **Coaches (n=25)** | | | **Medical Personnel (n=62)** | | | **Players (n=26)** | | | **Researchers (n=31)** | | | **All Participants (n=167)** | | |
| --- | --- | --- | --- | --- | --- | --- | --- | --- | --- | --- | --- | --- | --- | --- | --- |
|  | **1-3**  **Not important** | **4-6**  **Important** | **7-9**  **Very important** | **1-3**  **Not important** | **4-6**  **Important** | **7-9**  **Very important** | **1-3**  **Not important** | **4-6**  **Important** | **7-9**  **Very important** | **1-3**  **Not important** | **4-6**  **Important** | **7-9**  **Very important** | **1-3**  **Not important** | **4-6**  **Important** | **7-9**  **Very important** |
| **Data collection date** | 13% | 16% | 71% | 0% | 25% | 75% | 6% | 28% | 64% | 0% | 17% | 83% | 7% | 19% | 74% |
| **Country** | 21% | 31% | 58% | 0% | 28% | 72% | 9% | 35% | 56% | 0% | 24% | 76% | 9% | 24% | 67% |
| **Activity** | 4% | 8% | 88% | 0% | 3% | 97% | 0% | 12% | 88% | 0% | 2% | 98% | 1% | 6% | **93%** |
| **Tournament or league type** | 13% | 30% | 57% | 0% | 31% | 69% | 0% | 53% | 47% | 0% | 32% | 68% | 11% | 27% | 62% |
| **National or international** | 26% | 26% | 48% | 0% | 23% | 77% | 12% | 49% | 39% | 2% | 42% | 56% | 13% | 28% | 59% |
| **Playing level** | 4% | 4% | 92% | 0% | 3% | 97% | 6% | 15% | 79% | 0% | 0% | 100% | 3% | 4% | **93%** |
| **Number of players per activity** | 8% | 34% | 58% | 6% | 52% | 42% | 16% | 40% | 44% | 2% | 27% | 61% | 18% | 32% | 50% |
| **Number of players overall** | 9% | 5% | 86% | 3% | 9% | 88% | 3% | 13% | 84% | 2% | 5% | 93% | 4% | 8% | **88%** |
| **Number of Teams** | 17% | 8% | 75% | 2% | 7% | 81% | 3% | 16% | 81% | 2% | 15% | 83% | 5% | 14% | 81% |
| **Sex** | 8% | 4% | 88% | 0% | 2% | 98% | 0% | 12% | 88% | 0% | 0% | 100% | 2% | 3% | **95%** |
| **Age** | 0% | 7% | 83% | 0% | 5% | 95% | 0% | 27% | 73% | 0% | 5% | 95% | 0% | 11% | **89%** |
| **Age Group** | 0% | 7% | 83% | 2% | 15% | 83% | 6% | 28% | 66% | 0% | 12% | 88% | 2% | 17% | **81%** |
| **Type of header** | 4% | 6% | 80% | 2% | 12% | 86% | 3% | 12% | 85% | 0% | 34% | 66% | 4% | 16% | **80%** |
| **Level of Control** | 4% | 25% | 71% | 2% | 18% | 80% | 6% | 24% | 70% | 3% | 44% | 53% | 5% | 26% | 69% |
| **Attempted Header** | 46% | 37% | 17% | 10% | 48% | 42% | 16% | 49% | 35% | 15% | 65% | 20% | 30% | 38% | 32% |
| **Head injury** | 13% | 7% | 80% | 0% | 0% | 100% | 0% | 6% | 94% | 2% | 8% | 90% | 4% | 3% | **93%** |
| **Non-header related head impact** | 13% | 12% | 75% | 0% | 5% | 95% | 3% | 9% | 88% | 5% | 10% | 85% | 5% | 7% | **88%** |
| **Time between headers** | 8% | 29% | 63% | 2% | 32% | 66% | 0% | 19% | 81% | 5% | 49% | 44% | 7% | 30% | 63% |
| **Pitch location** | 21% | 21% | 58% | 5% | 32% | 63% | 10% | 53% | 37% | 2% | 57% | 41% | 16% | 33% | 51% |
| **Ball-to-head point of contact** | 4% | 33% | 63% | 2% | 15% | 83% | 0% | 12% | 78% | 2% | 37% | 61% | 6% | 21% | 73% |
| **Ball distance before header** | 4% | 33% | 63% | 0% | 30% | 70% | 6% | 16% | 78% | 5% | 39% | 54% | 9% | 24% | 67% |
| **Ball distance after header** | 17% | 33% | 50% | 2% | 39% | 59% | 13% | 37% | 50% | 5% | 63% | 32% | 14% | 37% | 49% |
| **Ball delivery type** | 8% | 28% | 64% | 3% | 27% | 70% | 3% | 42% | 55% | 2% | 32% | 66% | 9% | 26% | 65% |
| **Ball deliverer** | 82% | 6% | 12% | 81% | 2% | 17% | 81% | 5% | 14% | 81% | 4% | 15% | **81%** | 4% | 15% |
| **Angel of ball direction** | 16% | 32% | 52% | 3% | 34% | 63% | 0% | 34% | 66% | 8% | 72% | 20% | 10% | 39% | 51% |
| **Match time** | 29% | 38% | 33% | 2% | 42% | 56% | 22% | 59% | 19% | 10% | 70% | 20% | 26% | 38% | 36% |
| **Match score** | 80% | 5% | 15% | 81% | 14% | 5% | 85% | 9% | 6% | 82% | 6% | 12% | **85%** | 4% | 11% |
| **Playing position** | 8% | 32% | 60% | 2% | 28% | 70% | 6% | 28% | 64% | 2% | 27% | 71% | 5% | 28% | 67% |
| **Attack or defence** | 16% | 32% | 52% | 5% | 26% | 69% | 12% | 49% | 39% | 7% | 47% | 46% | 16% | 30% | 54% |
| **Possession** | 28% | 36% | 36% | 6% | 52% | 42% | 19% | 50% | 31% | 18% | 62% | 20% | 26% | 40% | 34% |
| **Elbow position** | 8% | 38% | 54% | 7% | 51% | 42% | 17% | 52% | 31% | 8% | 67% | 25% | 19% | 43% | 38% |
| **Player movement** | 0% | 12% | 88% | 3% | 22% | 75% | 3% | 29% | 68% | 5% | 46% | 49% | 6% | 26% | 68% |
| **Player movement direction** | 12% | 20% | 68% | 3% | 39% | 58% | 0% | 39% | 61% | 10% | 66% | 24% | 12% | 37% | 51% |
| **Jump height** | 16% | 32% | 52% | 3% | 45% | 52% | 9% | 44% | 47% | 12% | 76% | 12% | 20% | 37% | 40% |
| **Head height** | 16% | 48% | 36% | 6% | 44% | 50% | 10% | 53% | 37% | 13% | 77% | 10% | 21% | 44% | 35% |
| **Heading under pressure** | 12% | 24% | 64% | 3% | 30% | 67% | 0% | 27% | 73% | 10% | 61% | 29% | 11% | 31% | 58% |
| **Contest** | 8% | 20% | 72% | 2% | 12% | 86% | 0% | 19% | 81% | 5% | 45% | 51% | 6% | 20% | 74% |
| **Aerial duel** | 8% | 20% | 72% | 3% | 22% | 75% | 3% | 29% | 69% | 5% | 39% | 56% | 8% | 23% | 69% |
| **Physical duel** | 8% | 25% | 67% | 6% | 33% | 61% | 3% | 30% | 67% | 10% | 41% | 49% | 13% | 26% | 61% |
| **Body contact during duel** | 12% | 12% | 76% | 4% | 4% | 92% | 3% | 18% | 79% | 2% | 42% | 56% | 7% | 16% | 77% |
| **Protective body positioning** | 12% | 24% | 64% | 5% | 34% | 61% | 3% | 55% | 42% | 10% | 57% | 33% | 15% | 34% | 51% |
| **Player localisation** | 12% | 44% | 44% | 13% | 48% | 39% | 6% | 66% | 28% | 17% | 63% | 20% | 24% | 44% | 32% |
| **Consequence of header** | 8% | 36% | 58% | 3% | 22% | 75% | 3% | 34% | 63% | 8% | 36% | 56% | 8% | 27% | 65% |
| **Drill** | 17% | 25% | 58% | 2% | 31% | 67% | 12% | 46% | 42% | 5% | 46% | 49% | 11% | 32% | 57% |
| **Final Score** | 88% | 2% | 10% | 87% | 4% | 9% | 82% | 9% | 9% | 87% | 6% | 7% | **86%** | 6% | 8% |

Key:

Green: >80% to include

Pink: >80% to exclude

## Phase 1- Second-Round Questionnaire

Table 4 contains the results from the Phase 1- second round questionnaire for all participants and per role.

*Key:*

*Green: >80% to include*

*Pink: >80% to exclude*

*Blue: Delphi Steering Committee voted for inclusion*

### Table 4: Agreement results for Phase 1 - first-round questionnaire for all participants and per role (%)

| **Descriptor** | **Coaches (n=25)** | | | **Medical Personnel (n=62)** | | | **Players (n=26)** | | | **Researchers (n=31)** | | | **All Participants (n=144)** | | |
| --- | --- | --- | --- | --- | --- | --- | --- | --- | --- | --- | --- | --- | --- | --- | --- |
|  | **1-3**  **Not important** | **4-6**  **Important** | **7-9**  **Very important** | **1-3**  **Not important** | **4-6**  **Important** | **7-9**  **Very important** | **1-3**  **Not important** | **4-6**  **Important** | **7-9**  **Very important** | **1-3**  **Not important** | **4-6**  **Important** | **7-9**  **Very important** | **1-3**  **Not important** | **4-6**  **Important** | **7-9**  **Very important** |
| **Data collection date** | 0% | 9% | 91% | 2% | 10% | 89% | 0% | 8% | 92% | 3% | 3% | 94% | 1% | 8% | 91% |
| **Country** | 0% | 16% | 84% | 6% | 18% | 76% | 0% | 12% | 88% | 0% | 2% | 80% | 83% | 17% | 80% |
| **Tournament or league type** | 0% | 12% | 88% | 5% | 13% | 82% | 4% | 19% | 77% | 6% | 10% | 84% | 4% | 13% | 83% |
| **National or international** | 4% | 12% | 84% | 13% | 18% | 69% | 8% | 12% | 81% | 6% | 23% | 71% | 9% | 17% | 74% |
| **Number of players per activity** | 22% | 9% | 70% | 15% | 27% | 58% | 15% | 15% | 69% | 6% | 19% | 74% | 11% | 19% | 70% |
| **Level of Control** | 4% | 12% | 84% | 2% | 16% | 82% | 4% | 15% | 81% | 6% | 13% | 81% | 3% | 15% | 82% |
| **Attempted Header** | 20% | 32% | 48% | 43% | 43% | 15% | 27% | 31% | 42% | 20% | 53% | 27% | 31% | 41% | 28% |
| **Time between headers** | 0% | 29% | 71% | 2% | 28% | 70% | 4% | 19% | 77% | 3% | 13% | 84% | 2% | 23% | 75% |
| **Pitch location** | 4% | 29% | 67% | 16% | 19% | 65% | 8% | 19% | 73% | 6% | 19% | 74% | 10% | 21% | 69% |
| **Ball-to-head point of contact** | 0% | 16% | 84% | 3% | 16% | 81% | 4% | 8% | 88% | 3% | 0% | 97% | 3% | 11% | 86% |
| **Ball distance before header** | 4% | 17% | 79% | 5% | 11% | 84% | 0% | 15% | 85% | 0% | 23% | 77% | 3% | 15% | 82% |
| **Ball distance after header** | 12% | 28% | 60% | 2% | 41% | 57% | 8% | 15% | 77% | 3% | 45% | 52% | 5% | 35% | 60% |
| **Ball delivery type** | 0% | 16% | 84% | 6% | 24% | 69% | 8% | 19% | 73% | 3% | 13% | 84% | 5% | 19% | 76% |
| **Angel of ball direction** | 8% | 44% | 48% | 10% | 39% | 51% | 8% | 27% | 65% | 6% | 45% | 48% | 8% | 39% | 52% |
| **Match time** | 16% | 48% | 36% | 24% | 53% | 23% | 20% | 40% | 40% | 20% | 50% | 30% | 21% | 49% | 30% |
| **Playing position** | 0% | 16% | 84% | 5% | 15% | 81% | 4% | 12% | 85% | 3% | 16% | 81% | 3% | 15% | 83% |
| **Attack or defence** | 20% | 12% | 68% | 13% | 15% | 73% | 15% | 4% | 81% | 19% | 10% | 71% | 16% | 11% | 73% |
| **Possession** | 24% | 44% | 32% | 30% | 48% | 23% | 35% | 31% | 35% | 29% | 45% | 26% | 29% | 43% | 27% |
| **Elbow position** | 17% | 46% | 38% | 17% | 50% | 33% | 19% | 42% | 38% | 27% | 37% | 37% | 19% | 45% | 36% |
| **Player movement** | 0% | 28% | 72% | 5% | 11% | 84% | 0% | 8% | 92% | 0% | 19% | 81% | 2% | 15% | 83% |
| **Player movement direction** | 4% | 44% | 52% | 11% | 23% | 66% | 12% | 15% | 73% | 6% | 39% | 55% | 9% | 28% | 63% |
| **Jump height** | 13% | 46% | 42% | 21% | 31% | 48% | 23% | 38% | 38% | 13% | 42% | 45% | 18% | 37% | 44% |
| **Head height** | 13% | 63% | 25% | 20% | 48% | 33% | 27% | 42% | 31% | 23% | 43% | 33% | 21% | 48% | 31% |
| **Heading under pressure** | 4% | 8% | 88% | 11% | 11% | 77% | 12% | 15% | 73% | 10% | 13% | 77% | 9% | 11% | 80% |
| **Contest** | 0% | 8% | 92% | 3% | 10% | 87% | 0% | 4% | 96% | 6% | 3% | 90% | 3% | 7% | 90% |
| **Aerial duel** | 4% | 16% | 80% | 8% | 10% | 82% | 4% | 12% | 85% | 3% | 3% | 93% | 6% | 10% | 85% |
| **Physical duel** | 4% | 12% | 84% | 11% | 11% | 77% | 8% | 8% | 85% | 3% | 17% | 80% | 8% | 12% | 80% |
| **Body contact during duel** | 0% | 8% | 92% | 8% | 10% | 82% | 4% | 8% | 88% | 3% | 6% | 90% | 5% | 8% | 87% |
| **Protective body positioning** | 13% | 33% | 54% | 15% | 26% | 59% | 15% | 27% | 58% | 10% | 21% | 69% | 14% | 26% | 60% |
| **Player localisation** | 20% | 52% | 28% | 16% | 63% | 21% | 31% | 38% | 31% | 17% | 50% | 33% | 20% | 54% | 27% |
| **Consequence of header** | 4% | 28% | 68% | 10% | 11% | 79% | 12% | 0% | 88% | 3% | 6% | 90% | 8% | 11% | 81% |
| **Drill** | 4% | 32% | 64% | 7% | 21% | 72% | 4% | 19% | 77% | 7% | 23% | 70% | 6% | 23% | 71% |
| **Personal Protective Equipment** | 4% | 32% | 64% | 21% | 20% | 59% | 8% | 16% | 76% | 3% | 21% | 76% | 12% | 21% | 66% |
| **weather** | 16% | 52% | 32% | 27% | 37% | 35% | 20% | 36% | 44% | 17% | 40% | 43% | 22% | 40% | 38% |
| **Ball** | 16% | 32% | 52% | 8% | 23% | 69% | 8% | 27% | 65% | 6% | 29% | 65% | 9% | 27% | 64% |
| **Head movement** | 12% | 44% | 44% | 15% | 36% | 49% | 12% | 27% | 62% | 20% | 20% | 60% | 15% | 32% | 57% |
| **Head impact magnitude** | 9% | 41% | 50% | 16% | 24% | 60% | 4% | 29% | 67% | 4% | 18% | 79% | 10% | 26% | 63% |
| **Ball speed** | 17% | 26% | 57% | 10% | 29% | 61% | 12% | 20% | 68% | 0% | 17% | 83% | 9% | 24% | 66% |
| **Referee action** | 17% | 38% | 46% | 18% | 37% | 45% | 12% | 31% | 58% | 10% | 32% | 58% | 15% | 35% | 50% |
| **Player size** | 29% | 42% | 29% | 37% | 35% | 27% | 27% | 27% | 46% | 14% | 38% | 48% | 29% | 35% | 35% |
| **Playing surface** | 12% | 28% | 60% | 32% | 31% | 37% | 23% | 15% | 62% | 27% | 17% | 57% | 26% | 24% | 40% |
| **Player stability** | 13% | 29% | 58% | 21% | 29% | 50% | 17% | 21% | 63% | 10% | 29% | 61% | 16% | 28% | 56% |
| **Eye tracking** | 17% | 22% | 61% | 26% | 24% | 50% | 12% | 28% | 60% | 14% | 31% | 55% | 19% | 26% | 55% |
| **Player** | 25% | 46% | 29% | 49% | 21% | 30% | 29% | 25% | 46% | 34% | 38% | 28% | 38% | 30% | 32% |

Key :

Green: >80% to include

Pink: >80% to exclude

Blue: Delphi Steering Committee voted for inclusion

## Phase 2- First-Round Questionnaire

Phase 2 included two questionnaires, round-one and round-two. These questionnaires focused on achieving consensus on the definition of each descriptor included in Phase 1.

Table 5 contains the results from the Phase 2- first round questionnaire for all participants and per role.

Participants were able to suggest alternative definitions which are also included in this table, along with the role of the participant making the suggestion.

*Key:*

*Green: >80% to include*

### Table 5: Agreement results Phase 2- first round questionnaire for all participants and per role (%).

| **Descriptor and definition** | **Coaches**  **(n=21)** | **Medical Personnel**  **(n=57)** | **Players**  **(n=21)** | **Researchers**  **(n=22)** | **All Participants**  **(n=121)** |  |
| --- | --- | --- | --- | --- | --- | --- |
| **Data collection dates:**  The date, duration or period that data were collected | 95% | 98% | 95% | 100% | 98% |  |
| **Country:**  The country or countries where data were collected | 100% | 96% | 100% | 100% | 98% |  |
| **Activity type:**  The activity type that the data relates to | 90% | 95% | 90% | 95% | 93% |  |
| **Playing level:**  The playing level of the players that the data relates to | 90% | 95% | 90% | 95% | 93% |  |
| **Number of players (overall):**  The total number of players for whom data were collected | 100% | 96% | 100% | 100% | 98% |  |
| **Number of teams:**  The number of teams for whom data were collected | 100% | 98% | 100% | 100% | 99% |  |
| **Sex:**  The sex of the players for whom data were collected | 95% | 96% | 100% | 95% | 96% |  |
| **Age:**  The age of players for whom data were collected | 100% | 100% | 95% | 100% | 99% |  |
| **Age group:**  The age group or groups that the players compete in for whom data were collected | 100% | 100% | 100% | 86% | 98% |  |
| **Type of header:**  The type, purpose or intent of the header that is performed | 90% | 98% | 95% | 91% | 95% |  |
| **Controlled or uncontrolled header**  The level of control in the redirection of the ball that is demonstrated by the player performing the header | 81% | 96% | 90% | 91% | 92% |  |
| **Head injury:**  The header resulted in a potential head injury or diagnosed head injury  The header resulted in a player being unable to immediately resume play following the header.  The header resulted in an on-pitch or off-pitch medical assessment  The header resulted in a player being unable to immediately resume play following the header, and on or off-pitch medical assessment | 43%  14%  19%  19% | 40%  14%  11%  21% | 23%  14%  5%  48% | 38%  14%  19%  24% | 40%  13%  11%  27% |  |
|  | Suggested alternative definition (coach):  The header resulted in an injury or potential injury to the head which required (on or off-pitch) medical assessment | | | | | |
| **Non-header related head impact:**  A non-header related head impact | 100% | 93% | 90% | 100% | 95% |  |
| **Ball-to-head point of contact:**  The area of the head where the ball makes contact during the header | 100% | 96% | 100% | 100% | 98% |  |
| **Ball distance before header:**  The distance the ball travelled before it was headed | 95% | 93% | 95% | 100% | 95% |  |
| **Playing position:**  The tactical or field position that the player heading the ball was in at the time of the header  The position that the player heading the ball was in at the start of the match (i.e. the starting line-up), or when initially substituted into the match | 71%  29% | 56%  44% | 76%  24% | 64%  36% | 57%  43% |  |
| **Player movement:**  The movement of the player at the time of the header | 95% | 96% | 100% | 100% | 98% |  |
| **Heading under pressure:**  Whether the player completing the header was under direct, indirect or no pressure from an opponent player at the time of the header | 100% | 98% | 100% | 100% | 99% |  |
| **Contested nature of the header**  The header was performed during a contested (duel) or uncontested (no duel) situation  The header was performed in the absence (uncontested) or presence (contested) of an aerial or other physical duel | 71%  29% | 72%  28% | 57%  43% | 45%  55% | 65%  35% |  |
| **Aerial duel**  Two or more players competing for a ball that is above shoulder height; where at least one player is off the ground and is being physically challenged by an opposition player.  Two or more players competing for the header (with both players off the ground)  Two or more players competing for the header with at least one player jumping and one opponent within physical contact range (<1m) | 70%  15%  10% | 61%  20%  19% | 30%  43%  27% | 43%  29%  28% | 54%  25%  21% |  |
|  | Suggested alternative definition (coach): Two or more players competing for a ball that is above shoulder height; where at least one player is off the ground and is being physically challenged by an opposition player within physical contact range (< 1m). | | | | | |
| **Physical (header) duel**  Two or more players physically competing for a header and with all the players involved being grounded.  Two or more players from opposite teams are competing physically for a header to either win or retain possession of the ball for their team and all players are on the ground. The physical duel has to have started prior to any player receiving the ball. | 85%  15% | 75%  25% | 55%  45% | 71%  29% | 69%  31% |  |
|  | Suggested alternative definition (medical): Two or more players physically competing for a header with both players (if two players) or the majority of players involved (if more than two players) being grounded. | | | | | |
| **Body contact during a duel/header**  A situation where two or more players are competing for the header and the nature of the body contact observed between players  Physical contact between the player performing the header and any other player was observed. | 57%  43% | 53%  47% | 65%  45% | 41%  59% | 53%  47% |  |
|  | Suggested alternative definition (player): Physical contact between the player attempting to head the ball and any other player body part (e.g., head, shoulder, elbow) and/ or object (e.g., ground or goalpost) | | | | | |
| **Consequence of header**  The immediate outcome of the header  The possession outcome of a header performed by a player in, out or in contest for possession following a distribution, possession contest or defensive event. | 89%  11% | 80%  20% | 72%  18% | 77%  23% | 81%  19% |  |
| **National or international**  Whether data were collected during a national or international tournament or competition | 100% | 100% | 90% | 100% | 98% |  |
| **Number of players per activity type**  The number of players per team (for match data) or the number of players involved in each practice session or training activity | 100% | 100% | 95% | 95% | 98% |  |
| **Attempted header**  A player attempting to head the ball without contact being observed between the ball and the head | 100% | 98% | 95% | 100% | 100% |  |
| **Time between headers**  The time that has elapsed between headers for individual players | 100% | 93% | 100% | 95% | 96% |  |
|  | Suggested alternative definition (researcher): The time that has elapsed between two headers from the same player | | | | | |
| **Pitch location**  The location on the pitch where the header occurred | 100% | 95% | 100% | 100% | 98% |  |
| **Ball distance- after header**  The distance the ball travelled after it was headed until the ball either hit the ground, went out of play, scored a goal or was touched by another player | 95% | 96% | 100% | 95% | 97% |  |
| **Ball delivery type/situation**  The delivery type of the ball | 95% | 98% | 100% | 86% | 96% |  |
| **Ball**  Information about the ball being used in the match or training session | 90% | 100% | 100% | 95% | 98% |  |
| **Ball deliverer**  The shirt number of the player who played the ball immediately before the header  The position of the player who played the ball immediately before the header  The team of the player who played the ball immediately before the header  The position and team of the player who played the ball immediately before the header | 10%  15%  10%  65% | 18%  22%  15%  45% | 0%  23%  15%  62% | 9%  24%  29%  38% | 8%  21%  23%  48% |  |
| **Angle of ball direction**  The angle of flight of the ball | 100% | 96% | 100% | 100% | 98% |  |
| **Ball speed**  The speed of the ball immediately before and/or immediately after the header  The speed of the ball recorded 1 second before and/or 1 second after the header | 98%  2% | 80%  20% | 62%  38% | 86%  14% | 82%  18% |  |
| **Attack or defence**  The team of the player heading the ball were in an attacking phase or defensive phase at the time of the header  If the team of the player who headed the ball were in possession prior to the header, did the header occur in their own half or the opposition half of the pitch | 71%  29% | 85%  15% | 100%  0% | 77%  23% | 82%  18% |  |
| **Possession**  Whether the team of the player performing the header was in possession, out of possession or in contest, when the header was performed. | 100% | 96% | 100% | 95% | 98% |  |
| **Elbow position**  The elbow position of the player when performing the header  The elbow position of the player when performing the header. In the case of a contested header the elbow position of the opponent player will also be coded.  Elbows of the player (or players, if a contested header) are bent and raised up away from sides for protection, support, and balance. | 84%  3%  13% | 78%  12%  10% | 76%  2%  22% | 81%  17%  2% | 80%  9%  11% |  |
| **Player movement direction**  The movement of the player immediately before they performed the header relative to the ball  The movement of the player immediately before they performed the header | 66%  34% | 100%  0% | 78%  22% | 75%  25% | 81%  19% |  |
| **Jump height *(for jumping headers only).***  If the header was performed while the player heading the ball was jumping, the height of the jump is recorded in relation to the ground.  If the header was performed while the player heading the ball was jumping, the height of the jump is recorded in relation to the player closest to them. | 10%  90% | 12%  88% | 8%  92% | 18%  82% | 13%  87% |  |
|  | Suggested alternative definition (coach): If the header was performed while the player heading the ball was jumping, the height of the jump is recorded in relation to the player closest to them (contested headers only). | | | | | |
| **Head height**  The height of the player’s head when the ball makes contact relative to a normal standing position | 100% | 95% | 100% | 95% | 98% |  |
| **Protective body positioning**  The presence of protective body positioning during a contested header | 100% | 95% | 100% | 100% | 99% |  |
| **Player localisation**  The position of the player heading the ball relative to the nearest other player at the time of the header  The position of the player heading the ball relative to the nearest opposition player at the time of the header | 92%  8% | 94%  6% | 91%  9% | 98%  2% | 95%  5% |  |
| **Head covering or personal protective equipment**  The player performing the header was observed to be wearing something that covered all or part of their head or face at the time of the header or a form of personal protective equipment | 100% | 100% | 100% | 100% | 100% |  |
| **Head movement**  The movement direction of the player’s head immediately before and/or after they completed the header | 100% | 90% | 100% | 100% | 98% |  |
| **Head impact magnitude**  The linear, rotational acceleration and/or angular velocity recorded during the header  The resultant linear and/or rotational acceleration recorded | 81%  19% | 73%  27% | 76%  24% | 50%  50% | 72%  18% |  |
|  | Suggested alternative definition (researcher): The peak resultant linear acceleration, rotational acceleration and/or rotational velocity computed from an objective measurement device (such as head impact sensor, instrumented mouthguard or inertial measurement unit) during the header | | | | | |
| **Player size**  The relative size of the player performing the header | 95% | 95% | 95% | 82% | 93% |  |
|  | Suggested alternative definition (researcher): For contested headers only: Relative size of player performing header (in relation to the other player they are in contest with) | | | | | |
| **Player stability**  How balanced the player heading the ball was at the time of the header | 100% | 96% | 100% | 100% | 98% |  |
|  | Suggested alternative definition (medical): The player heading the ball was stable or not stable when heading the ball. | | | | | |
| **Ball tracking**  Whether the player heading the ball had their eyes open and their head turned towards the direction of the in-coming ball immediately prior to the header.  Whether the player was tracking the in-coming ball immediately prior to the header.  Whether the player heading the ball had their eyes open immediately prior to the header. | 8%  82%  10% | 4%  92%  4% | 6%  89%  5% | 2%  95%  3% | 5%  90%  5% |  |
| **Match time**  The match time when the header occurred | 100% | 100% | 95% | 100% | 99% |  |
| **Weather**  The weather at the time of the header  The weather at the start of play | 91%  9% | 87%  13% | 100%  0% | 86%  14% | 89%  11% |  |
| **Referee**  The action of the referee in response to the heading event | 100% | 98% | 100% | 100% | 99% |  |
| **Playing surface**  The specific playing surface of the pitch | 100% | 100% | 95% | 100% | 99% |  |

*Key:*

*Green: >80% to include*

## Phase 2- Phase 2- Second Round Questionnaire

Table 6 contains the results from the Phase 2- second round questionnaire for all participants and per role.

*Key:*

*Green: >80% to include*

### Table 6: Agreement results Phase 2 - second-round questionnaire for all participants and per role (%).

| **Descriptor and definition** | **Coaches**  **(n=19)** | **Medical Personnel**  **(n=51)** | **Players**  **(n=14)** | **Researchers**  **(n=18)** | **All Participants**  **(n=102)** |
| --- | --- | --- | --- | --- | --- |
| **Head injury:**  The header resulted in a potential head injury or diagnosed head injury  The header resulted in a player being unable to immediately resume play following the header.  The header resulted in an on-pitch or off-pitch medical assessment  The header resulted in a player being unable to immediately resume play following the header, and on or off-pitch medical assessment  The header resulted in an injury or potential injury to the head which required (on or off-pitch) medical assessment | 77%  0%  2%  2%  19% | 67%  2%  2%  6%  23% | 88%  2%  2%  0%  8% | 72%  3%  0%  3%  12% | 78%  3%  2%  4%  13% |
| **Playing position:**  The tactical or field position that the player heading the ball was in at the time of the header  The position that the player heading the ball was in at the start of the match (i.e. the starting line-up), or when initially substituted into the match | 74%  26% | 83%  17% | 100%  0% | 87%  13% | 83%  17% |
| **Contested nature of the header**  The header was performed during a contested (duel) or uncontested (no duel) situation  The header was performed in the absence (uncontested) or presence (contested) of an aerial or other physical duel | 77%  23% | 90%  10% | 91%  9% | 100%  0% | 91%  9% |
| **Aerial duel**  Two or more players competing for a ball that is above shoulder height; where at least one player is off the ground and is being physically challenged by an opposition player.  Two or more players competing for the header (with both players off the ground)  Two or more players competing for the header with at least one player jumping and one opponent within physical contact range (<1m)  Two or more players competing for a ball that is above shoulder height; where at least one player is off the ground and is being physically challenged by an opposition player within physical contact range (< 1m). | 78%  0%  0%  22% | 78%  2%  1%  19% | 84%  1%  1%  14% | 84%  0%  0%  16% | 80%  1%  1%  18% |
| **Physical (header) duel**  Two or more players physically competing for a header and with all the players involved being grounded.  Two or more players from opposite teams are competing physically for a header to either win or retain possession of the ball for their team and all players are on the ground. The physical duel has to have started prior to any player receiving the ball.  Two or more players physically competing for a header with both players (if two players) or the majority of players involved (if more than two players) being grounded. | 68%  1%  31% | 79%  2%  19% | 72%  0%  31% | 91%  0%  9% | 80%  2%  18% |
| **Body contact during a duel/header**  A situation where two or more players are competing for the header and the nature of the body contact observed between players  Physical contact between the player performing the header and any other player was observed.  Physical contact between the player attempting to head the ball and any other player body part (e.g., head, shoulder, elbow) and/ or object (e.g., ground or goalpost) | 65%  14%  31% | 60%  18%  22% | 88%  3%  9% | 86%  12%  2% | 78%  6%  16% |
| **Time between headers**  The time that has elapsed between headers for individual players  The time that has elapsed between two headers from the same player | 76%  24% | 83%  17% | 82%  8% | 91%  9% | 85%  15% |
| **Ball deliverer**  The shirt number of the player who played the ball immediately before the header  The position of the player who played the ball immediately before the header  The team of the player who played the ball immediately before the header  The position and team of the player who played the ball immediately before the header | 4%  16%  13%  67% | 3%  2%  16%  79% | 0%  3%  3%  94% | 2%  0%  15%  83% | 3%  6%  10%  81% |
| **Jump height *(for jumping headers only).***  If the header was performed while the player heading the ball was jumping, the height of the jump is recorded in relation to the ground.  If the header was performed while the player heading the ball was jumping, the height of the jump is recorded in relation to the player closest to them.  If the header was performed while the player heading the ball was jumping, the height of the jump is recorded in relation to the player closest to them (contested headers only). | 4%  6%  90% | 6%  1%  93% | 11%  0%  89% | 12%  1%  87% | 6%  3%  91% |
| **Head impact magnitude**  The linear, rotational acceleration and/or angular velocity recorded during the header  The resultant linear and/or rotational acceleration recorded  The peak resultant linear acceleration, rotational acceleration and/or rotational velocity computed from an objective measurement device (such as head impact sensor, instrumented mouthguard or inertial measurement unit) during the header | 6%  0%  94% | 8%  1%  91% | 19%  0%  81% | 14%  1%  85% | 11%  1%  88% |
| **Player size**  The relative size of the player performing the header  For contested headers only: Relative size of player performing header (in relation to the other player they are in contest with) | 91%  9% | 93%  7% | 88%  12% | 94%  6% | 92%  8% |
| **Player stability**  How balanced the player heading the ball was at the time of the header  The player heading the ball was stable or not stable when heading the ball. | 72%  28% | 87%  13% | 69%  31% | 89%  11% | 81%  19% |

## Phase 3- First round questionnaire

Phase 3 only included one questionnaire (although a second-round questionnaire was possible, if needed). This questionnaire focused on the methods and reporting options for each descriptor from Phase 1.

Table 7 contains the results from the Phase 3 questionnaire for all participants and per role. The list number refers to the list of descriptors as per manuscript Table 2. Participants were asked whether they agreed with each list of descriptors, definitions and reporting options, with an open box to add comments or suggested modifications to any item. Thus, the percentage below indicates the proportion of participants who agreed with each list.

### Table 7: Completion of Phase 3 questionnaire for all participants and per role (%)

| **List** | **Coaches**  **(n=19)** | **Medical Personnel**  **(n=54)** | **Players**  **(n=19)** | **Researchers**  **(n=23)** | **All Participants**  **(n=115)** |
| --- | --- | --- | --- | --- | --- |
| **List one** | 100% | 93% | 95% | 91% | 94% |
| **List two** | 100% | 98% | 100% | 100% | 99% |
| **List three** | 95% | 93% | 95% | 91% | 93% |

### Suggested modifications

A number of participants suggested modifications to the reporting options.

List one:

Medical personnel “Playing level: Amateur: a player who plays organised football at club representative level but does not meet the criteria of a professional Recreational: (such as school, local club at or employee tournaments)”

Medical personnel: playing level: add “within a national league system” to option ii- amateur

List two:

No modifications were suggested.

List three:

Three participants (one player, one medical personnel, one researcher) suggested adding “no referee action” to the referee descriptor.
