# Supplementary material for: Consensus on the descriptors, definitions, and reporting methods for heading in football studies: A Delphi study
Source: PLoS One. 2025 Jul 17;20(7):e0327189. doi: 10.1371/journal.pone.0327189 (PMC12270182; doi:10.1371/journal.pone.0327189)
Supplement: Attachment — Submitted filename: Response to reviewers.docx [file pone.0327189.s003.docx]

10th June 2025

Dear Prof Lopes,

**Re: PONE-D-25-11018**

**Consensus on the descriptors, definitions, and reporting methods for heading incidence studies:  A Delphi study**

Thank you for your email dated 22nd May 2025.

Please find below Editor and Reviewer comments along with our response and an outline of any changes we have made to the revised manuscript.

**Journal requirements:**

1. When submitting your revision, we need you to address these additional requirements.

Please ensure that your manuscript meets PLOS ONE's style requirements, including those for file naming. The PLOS ONE style templates can be found at

<https://journals.plos.org/plosone/s/file?id=wjVg/PLOSOne_formatting_sample_main_body.pdf> and

<https://journals.plos.org/plosone/s/file?id=ba62/PLOSOne_formatting_sample_title_authors_affiliations.pdf>

**Author response**

Thank you. We have checked adherence to the journal requirements, including making any necessary edits such as removing the subheadings from the abstract, and ensuring the heading and sub-heading are in the correct format.

**Journal requirements**

2. Thank you for stating in your Funding Statement:

This project was part funded by a Thompson Equity Prize (The University of Sydney) awarded to KP.

Please provide an amended statement that declares *all* the funding or sources of support (whether external or internal to your organization) received during this study, as detailed online in our guide for authors at <http://journals.plos.org/plosone/s/submit-now>.  Please also include the statement “There was no additional external funding received for this study.” in your updated Funding Statement.

Please include your amended Funding Statement within your cover letter. We will change the online submission form on your behalf.

**Author response**

In response to this point and point 3 below, we have amended the funding section to read:

”This project was part funded by a Thompson Equity Prize (The University of Sydney) awarded to KP. KP, AS, TM, TEA, VG are all employed by non-profit football organisations and contributed to this study within the scope of their salaried roles. The specific roles of these authors are articulated in the ‘author contributorship’ section.” There was no additional external funding received for this study.”

**Journal requirements**

3. Thank you for stating the following in the Competing Interests section:

AS and KP declare employment with FIFA. KP and JG have been contracted Injury Spotters for FIFA organised tournaments (2023). KP, TM, VG and AS are members of FIFA’s Heading Expert Group. TM is chairman of UEFA´s and the German FA´s (DFB) Medical Committee as well as of the German League Organisation´s (DFL) working group “Medicine in Professional Football”. TEA is Chief Medical Officer of the Football Association of Norway (NFF). VG is the Chief Medical Officer at FIFPRO. All authors declare no other relevant financial or non-financial competing interests.

We note that one or more of the authors are employed by a commercial company.

a. Please provide an amended Funding Statement declaring this commercial affiliation, as well as a statement regarding the Role of Funders in your study. If the funding organization did not play a role in the study design, data collection and analysis, decision to publish, or preparation of the manuscript and only provided financial support in the form of authors' salaries and/or research materials, please review your statements relating to the author contributions, and ensure you have specifically and accurately indicated the role(s) that these authors had in your study. You can update author roles in the Author Contributions section of the online submission form.

Please also include the following statement within your amended Funding Statement.

“The funder provided support in the form of salaries for authors, but did not have any additional role in the study design, data collection and analysis, decision to publish, or preparation of the manuscript. The specific roles of these authors are articulated in the ‘author contributions’ section.”

If your commercial affiliation did play a role in your study, please state and explain this role within your updated Funding Statement.

**Author response**

Please refer to point 2 above for our updated funding statement. We also wish to confirm that none of the authors are employed by a commercial company as suggested. All authors are either affiliated with a university or a non-profit organisation (this includes FIFA- a non-profit organisation under Swiss Law, FIFPRO- football players’ union, plus Union of European Football Associations (UEFA), German Football Association, German League, Football Association of Norway are also all non-profit organisations).

**Journal requirements**

b. Please also provide an updated Competing Interests Statement declaring this commercial affiliation along with any other relevant declarations relating to employment, consultancy, patents, products in development, or marketed products, etc.

Within your Competing Interests Statement, please confirm that this commercial affiliation does not alter your adherence to all PLOS ONE policies on sharing data and materials by including the following statement: "This does not alter our adherence to  PLOS ONE policies on sharing data and materials.” (as detailed online in our guide for authors <http://journals.plos.org/plosone/s/competing-interests>) . If this adherence statement is not accurate and  there are restrictions on sharing of data and/or materials, please state these. Please note that we cannot proceed with consideration of your article until this information has been declared.

Please include both an updated Funding Statement and Competing Interests Statement in your cover letter. We will change the online submission form on your behalf.

**Author response**

We have also updated the competing interest statement as follows:

“**Competing interests**

KP, AS, TM, TEA, VG are all currently employed by non-profit football organisations. AS and KP declare employment with FIFA. TM is chairman of UEFA´s and the German FA´s (DFB) Medical Committee as well as of the German League Organisation´s (DFL) working group “Medicine in Professional Football”. TEA is Chief Medical Officer of the Football Association of Norway (NFF). VG is the Chief Medical Officer at FIFPRO.JG has been a paid contracted Injury Spotter for FIFA organised tournaments (2023). TM and VG are non-paid members of FIFA’s Heading Expert Group. This does not alter our adherence to PLOS ONE policies on sharing data and materials. All authors declare no other relevant financial or non-financial competing interests.”

**Journal requirements**

4. We note that this data set consists of interview transcripts. Can you please confirm that all participants gave consent for interview transcript to be published?

If they DID provide consent for these transcripts to be published, please also confirm that the transcripts do not contain any potentially identifying information (or let us know if the participants consented to having their personal details published and made publicly available). We consider the following details to be identifying information:

- Names, nicknames, and initials

- Age more specific than round numbers

- GPS coordinates, physical addresses, IP addresses, email addresses

- Information in small sample sizes (e.g. 40 students from X class in X year at X university)

- Specific dates (e.g. visit dates, interview dates)

- ID numbers

Or, if the participants DID NOT provide consent for these transcripts to be published:

- Provide a de-identified version of the data or excerpts of interview responses

- Provide information regarding how these transcripts can be accessed by researchers who meet the criteria for access to confidential data, including:

a) the grounds for restriction

b) the name of the ethics committee, Institutional Review Board, or third-party organization that is imposing sharing restrictions on the data

c) a non-author, institutional point of contact that is able to field data access queries, in the interest of maintaining long-term data accessibility.

d) Any relevant data set names, URLs, DOIs, etc. that an independent researcher would need in order to request your minimal data set.

For further information on sharing data that contains sensitive participant information, please see: [https://journals.plos.org/plosone/s/data-availability#loc-human-research-participant-data-and-other-sensitive-data](https://journals.plos.org/plosone/s/data-availability#loc-human-research-participant-data-and-other-sensitive-data)

If there are ethical, legal, or third-party restrictions upon your dataset, you must provide all of the following details ([https://journals.plos.org/plosone/s/data-availability#loc-acceptable-data-access-restrictions](https://journals.plos.org/plosone/s/data-availability#loc-acceptable-data-access-restrictions)):

a) A complete description of the dataset

b) The nature of the restrictions upon the data (ethical, legal, or owned by a third party) and the reasoning behind them

c) The full name of the body imposing the restrictions upon your dataset (ethics committee, institution, data access committee, etc)

d) If the data are owned by a third party, confirmation of whether the authors received any special privileges in accessing the data that other researchers would not have

e) Direct, non-author contact information (preferably email) for the body imposing the restrictions upon the data, to which data access requests can be sent

**Author response**

We can confirm that the manuscript does NOT contain any data derived from participant interviews. All the data were provided via the REDCap survey instrument, which was distributed via email to participants. The complete dataset is included in the Supplementary Appendix.

**Journal requirements**

5. When completing the data availability statement of the submission form, you indicated that you will make your data available on acceptance. We strongly recommend all authors decide on a data sharing plan before acceptance, as the process can be lengthy and hold up publication timelines. Please note that, though access restrictions are acceptable now, your entire data will need to be made freely accessible if your manuscript is accepted for publication. This policy applies to all data except where public deposition would breach compliance with the protocol approved by your research ethics board. If you are unable to adhere to our open data policy, please kindly revise your statement to explain your reasoning and we will seek the editor's input on an exemption. Please be assured that, once you have provided your new statement, the assessment of your exemption will not hold up the peer review process.

**Author response**

We apologise for any confusion. All the data from this study are included in Supplementary Appendix including the breakdown of responses for each questionnaire. We hope this appendix will be publicly accessible along with the manuscript (if accepted for publication).

**Journal requirements**

6. Your ethics statement should only appear in the Methods section of your manuscript. If your ethics statement is written in any section besides the Methods, please delete it from any other section.

**Author response**

We can confirm that our ethics statement only appears in the Methods section of the manuscript. We have deleted it from the title page.

**Additional Editor Comments:**
The reviewers have made interesting comments that will help enhance your manuscript. Please follow closely their recommendations.

**Author response**

We thank the reviewers for their considered and valued feedback. We have addressed each comment below.

**Reviewer 1 comments**

1. This original research paper describes a Delphi study to develop a method of develop a definition of ‘heading of the ball’ (i.e., heading) in soccer and a protocol to assess the skill characteristics for the purposes of injury surveillance and technique analysis. The protocol is designed to be applied primarily to video, but other data sources (e.g., accelerometery) are also included. As far as I can determine, the study has not been published elsewhere.

   The participants and methods used for the Delphi study are appropriate, largely concordant with published guidelines (ACCORD and CREDES) and reported in sufficient detail. One hundred and sixty-seven 167 participants with experience in research and/or soccer who commenced the Delphi study. The Delphi study consisted of five rounds over three phases to select the characteristics that should comprise the minimum analysis, and determine definitions. The first phase determined the fields (described as descriptors) for the analysis, the second phase defined the fields while the final phase determined the method or list of field variables used to assess/describe each field. Of the five rounds of consensus, 50% of participants completed all 5 rounds. Although the attrition is of some concern, 80+ participants is still substantial and efforts are made to report on the characteristics of respondents at each step. Subsequently, 27 fields were selected for inclusion in the minimal analysis and a further 27 characteristics, deemed not as important by participants in the Delphi study, were also defined for an expanded analysis.

   The described protocol will provide a standardised approach to identifying a header and investigating characteristics of heading in soccer from video, allowing the incidence of heading and the characteristics analysed to be determined and, when combined with injury surveillance data, potentially allowing for causal inference. Whilst there are some characteristics which I expect will be challenging to evaluate reliably from video, this is beyond the scope of the current study and the authors have wisely encouraged reporting of reliability statistics. It is my opinion that the manuscript meets the aims and scope of PLoSOne and would be of interest to its readers, subject to consideration of several revisions.

**Author response**

We thank the reviewer for this positive summary. We agree that the response rate for participants who completed all 5 questionnaires was lower than we had hoped for at 53%, however, the response rate for each questionnaire (excluding the first questionnaire) ranged from 61-86%, with 89% of participants completing at least 2 questionnaires (with many completing the first, second and fifth-final questionnaires).

**Reviewer comment**

2. Firstly, I would encourage the authors to thoroughly review and edit the manuscript to improve its readability. There were many instances where the language used is unclear. I provide three of the many examples throughout the manuscript:

“Heading incidence research could be explored as one approach of assessing the potential causal inference between heading and long-term issues with brain health” - Does you mean that you wish to explore heading incidence research or that you want to investigate the incidence of heading and heading characteristics? I believe it's the latter but the expression is overly complex.

**Author response**

Thank you for this comment. We have thoroughly reviewed the manuscript and made a number of edits to improve its readability. It was a complex process to convey, therefore, we appreciate this additional feedback.

We have amended this sentence to now read:

“Heading in football research, which includes the incidence and characteristics of headers, could be explored as one approach of assessing the potential causal inference between headers and long-term issues with brain health.”

**Reviewer comment**

3. Similarly, "our understanding of heading risk then THIS needs" (lines122-3). What do you mean by THIS?, please be specific so that it is unambiguous for the reader. Finally "Research methods experts, having... consensus projects" (lines 139-40) could be simplified as "Delphi and consensus study experts", or similar. Please be clear and explicit throughout.

**Author response**

Thank you for these comments. The first sentence has been amended to:

“However, if heading in football research is to contribute to our understanding of the potential relationship between header exposure and short-, medium, or long-term brain health of football players then the collection of header exposure data needs to be of the highest quality possible.”

And the second sentence has been amended to:

“Professionally, 2 members were Delphi and consensus study experts, and 6 members had health, and health related research backgrounds.”

Other edits have also been made in response to this feedback and feedback from reviewer 2, which have been made to the manuscript using tracked changes.

**Reviewer comment**

4. Additionally, there are many typos throughout which need correction (e.g., “insures” vs ensures (line 116) or “can” vs could (line 118), “whereby” vs thereby (line 441), the repeated phrase on line 468, etc.).

**Author response**

Thank you for this comment. We apologise for any typos. The whole manuscript has been reviewed and typographical errors corrected.

**Reviewer comment**
5. Secondly, the introduction should be expanded to better position this work with respect to previous literature. The authors could better introduce the discussion in the field regarding ball heading and its long-term health impacts and define also define the difference between heading and non-heading impacts. From the context and literature, I suspected non-heading impacts may refer to be head-to-head contact and it wasn’t clear until very late in the manuscript that this wasn’t the case. The introduction could also briefly introduce efforts from soccer and other sports to define injury events and develop qualitative analysis methods (e.g., Tenga’s work, volleyball, tackling in rugby union etc.), what was good and what could be improved. This would then allow a discussion point on how this study improved on earlier studies, thus broadening the relevance of this study beyond heading incidence.

**Author response**

We tried to keep the introduction as brief as possible given the extended word count of the full manuscript. However, we have made a number of changes to the introduction, particularly with the inclusion of the following sentences to explain the long-term risk between football participation and brain health.

“Heading in football (soccer) research, which includes the incidence and characteristics of headers, could be explored as one approach of assessing the potential causal inference between header exposure and long-term issues with brain health in retired players. A recent systematic review and meta-analysis exploring the risk of football players in developing any neurodegenerative disease (including Alzheimer’s disease and other dementias, motor neurone disease, amyotrophic lateral sclerosis, and Parkinson’s disease) compared to general populations from nine included studies reported an odds ratio of 1.69 (95% CI 1.11-2.58) with significant heterogeneity between studies.(2) However, header, head impact, and/or head injury exposure was either not objectively collected or completely absent as an outcome measure in these studies, with causal inferences often based on career length,(3) and playing position (out-field versus goal keeper).(1)”

We have also added the following sentence regarding the difference between headers and unintentional ball-to-head impacts but we feel introducing further discussion regarding injury events in football or any other sport might detract from the main purpose of our study which is related to the skill of heading (and how this can be characterised and quantified) rather than head injuries in football:

“A recent systematic review highlighted several shortcomings related to current heading in football research, most importantly the lack of a standardised operational definition of a header, with some studies making no distinctions between a deliberate head-to-ball contact (i.e. a header), and an accidental or unintentional ball-to-head impact (such as a player being hit in the face by a ball delivered at close range).(5) This distinction is important given that higher head impact forces have been measured when the ball contact is unexpected.(8)”

**Reviewer comment**

6. Thirdly, relating to clarity of reporting, I note that the project was part funded through an academic prize at an Australian university although ethics approval was obtained in Switzerland. Were there other sources of funding (e.g., FIFA) which should be included?

**Author response**

Thank you, we have amended our funding section in response to the journal requirements as listed above.

**Reviewer comment**

7. The direction to include reliability in studies reporting incidence with the protocol is a good one. There are several instances where Cohen’s Kappa is not appropriate, such as continuous data, multiple raters (i.e., > 2) or where one variable occurs at a substantially higher frequency than others. For this reason, the authors might consider something like “agreement of >0.80 with an appropriate agreement statistic (e.g., cohen’s Kappa, Fleiss Kappa, ICC, percentage agreement, etc.)”

**Author response**

Thank you for this suggestion, we have amended this sentence to read:

“For minimum reporting descriptors which are not found to have a substantial level of inter-/intra-rater agreement with an appropriate agreement statistic (e.g., Cohen’s Kappa, Fleiss Kappa, Intraclass Correlation Coefficient >0.60) analysts may wish to combine certain reporting options to enhance reliability.

**Reviewer comment**

8. Finally, the reason for hijab being bracketed with protective equipment seems unusual and needs to be better explained to the reader. The other items listed on page 16 seem have an obvious relationship with skill and performance and so need little justification. Head covering on the other hand states that participants suggested that it be included but why it is important wasn’t mentioned in this article or in the associated systematic review. Did the expert panel form the view that a hijab attenuated head force and reduce the risk of injury or might obscure vision and increase the risk of injury etc?

**Author response**

Thank you for highlighting this. This was a descriptor that was suggested by one of the participants, so it is worded in the manuscript to identically match the wording used in the questionnaire. Head covering was grouped with personal protective equipment that was applied to the head as sometimes the reason for a head covering is not always clear.

**Reviewer 2 comments:**

Firstly, I would like to congratulation the authors for undertaking a comprehensive Delphi study. A strength of this study is the inclusion of an large number of participants.

**Author response**

Thank you for this positive feedback.

**Reviewer comment**
1. My major comment/concern is regarding the focus on ‘incidence’ – but then limited explanation of how one should accurately and robustly collect exposure. Given exposure time is the denominator for incidence, this will have a large impact. Especially in training – exposure could be continuous (start – end), drill duration (removal of ‘dead time’) or player involvement in the drills. I think it is the authors either point the reader to where this exists, and recommend the use of an existing definition, or include this.

**Author response**

Thank you for this comment, we have added the following sentences to the discussion:

**“**While it was outside of the scope of this study to report on the best approach to calculate incidence rates, incidence rates of headers have been presented using match exposure time based on earlier published formulas (number of headers/match exposure time) × 1000), with similar calculations for headers during training.(20,21)”

**Reviewer comment**

2. I do wonder if this is more than incidence – for examples some of definitions are beyond heading incidence. Isn’t heading incidence only one part of the story, especially when considering the results of this study even say that magnitude is important. Therefore, isn’t this a consensus for heading studies?

**Author response**

Thank you for this comment. We have amended the manuscript throughout to make it clearer that we are talking about heading in football studies, which includes both the incidence and characteristics of headers. We have also added a definition of heading as follows to make this clearer:

”For this study, heading was defined as a player performing a deliberate movement to redirect the trajectory of a ball using the head. Therefore, heading could result in either a header (with head-to-ball contact) or an attempted header (without head-to-ball contact), and could be performed in either a contested (duel) or uncontested (no duel) situation.”

**Reviewer comment**

3. I was also surprised that a head-head collision (during a header) wasn’t included. I’m not sure if this was deliberately excluded (based on the study design), or no one thought this was important, but it does feel like it warrants more discussion. E.g., if we know the head-head collision rates during different heading activities / in different cohorts – that is very helpful when considering ‘short-term’ head injury risk? This was discussed briefly in lines 476-478, and 540-543, but it seems an omission.

**Author response**

Head-to-head impact is included in the following descriptor and definition in Table 2:

| Non-header related head impact: | A head impact that is not classified as a header. | To be reported using the following options (NB more than one option might apply):  i. Unintentional ball-to-head impact: any physical contact between a player’s head and the ball, which is not considered deliberate (for example, ball impact with a player’s face or head when the ball is played at short range or ball impact to the back of a player’s head).  ii. Head impact - other player: any physical contact between a player’s head or face and another player’s body part (for example, head-to-head, elbow-to-head impact)  iii. Head impact – object: any physical contact between a player’s head or face and an object (such as ground-to-head, goalpost-to-head impact). |
| --- | --- | --- |

**Reviewer comment**

4. My minor comments are;

At first mention (in abstract and in main documents), report ‘football (i.e., soccer)’

**Author response**

Thank you, we have made this addition to the abstract and introduction.

**Reviewer comment**

5.You report short and long-term risk in abstract, but don’t define them in the introduction. I would suggest reporting the ‘short-, medium-, and longer-term risk’…. (and defining).

**Author response**

Thank you, we have added the following sentences to the introduction:

”Furthermore, objectively collected header exposure data can assist when exploring the potential short-term (defined as a single session of headers) (6), or medium-term (which includes header exposure over multiple practice sessions or across a season/s in active players) risk to player’s brain health. Exploring the potential short- and medium-term risk associated with repeated headers offer an alternative study design given the infeasibility of completing a randomised clinical trial in which young players are assigned to play football with or without heading with their cognitive function monitored over many decades.(7)”

**Reviewer comment**

6. Key points – include clarify what is the denominator for calculating 1000 hours.

**Author response**

Please see our response to point 1 above.

**Reviewer comment**

7. Key points (line 108). ‘Furthermore’, not ‘further’.

**Author response**

Thank for the suggestion. We have made this edit.

**Reviewer comment**

8. Introduction (line 119). I’m not sure what is encouraging? I couldn’t follow what ‘This is’ was referring to.

**Author response**

Thank you, this sentence has now been deleted as part of the overall changes to the introduction.

**Reviewer comment**
9. Introduction (line 124). A recent systematic review needs a reference

**Author response**

Our apologies, the reference was at the end of the next sentence. We have now added it to this sentence as well.

**Reviewer comment**
10. Method – I didn’t find the method very clear early on, and think a study design or overview would be helpful. For example, the ‘each objective and phase (1-3) on line 176 came out of the blue, and I didn’t know what each objective was, as the objective (singular – not pleural) on line 129-131 is set out as one objective. Figure 1 needs to do a better job of clarifying what the three phases are – which are nicely summarised on line 177-179.

**Author response**

We have re-drafted figure 1 to hopefully make this clearer and better reflect the narrative summary of the methods.

**Reviewer comment**
11. Method (line 153). In the journal information, you report University of Sydney as the ethics organisation, then Swiss Association…. In the main text. Please clarify.

**Author response**

Apologies, this was an editing error. To confirm, an ethics exemption was provided by Swiss Ethics. A copy of this has been provided to the journal, and any reference to the University of Sydney should have been deleted.

**Reviewer comment**
12. Results (line 315) The abbreviations (e.g., CAF, CONCACAF) on line 315 come before they are written in full (lines 330-333).

**Author response**

Thank you, we have now written these abbreviations out in full where they first appear.

**Reviewer comment**
13. Results (lines 326-328) The percentages reported add up to 99%

**Author response**

Yes, this missing 1% is due to rounding down in many of the individual percentages.

**Reviewer comment**
14. Table 1. The numbers (112, 67%) in the last row of the men or women column, don’t follow what were previous reported, therefore aren’t intuitive. It would be useful to report men and women?

**Author response**

Thank you for highlighting this. The last cell was missing the total for women. We have also added (M) next to the data for men, and (W) next to the data for women to make this clearer.

**Reviewer comment**
15. Limitations (line 474, 516, 529). ‘Furthermore’, not ‘further’.

**Author response**

This edit has been made.

**Reviewer comment**
16. Discussion (line 507) expand on how to capture exposure as the denominator

**Author response**

Please see our response to point 1 above.

We hope that our response has adequately addressed all comments and to everyone’s satisfaction.

We look forward to hearing from you soon,

Kind regards,

The Author Team
